# Kirigami-inspired metamaterials for programming constitutive laws: Mixed-mode multidirectional auxeticity and contact-induced stiffness modulation

**DOI:** 10.1016/j.isci.2022.105656

**Published:** 2022-12-08

**Authors:** Aryan Sinha, Tanmoy Mukhopadhyay

**Affiliations:** 1 Department of Aerospace Engineering, Indian Institute of Technology Kanpur, Kanpur, India

**Keywords:** Mechanics of materials, Mechanical property, Metamaterials

## Abstract

Stress-strain constitutive relations and Poisson’s ratios are fundamental properties of naturally occurring materials, based on which their mechanical applications can be designed. The lack of tailorability and restricted margin for such critical properties severely limit the bounds of achievable multi-functional engineering designs using conventional materials. Through analytical and numerical analyses, supported by elementary-level physical experiments, we have proposed a kirigami-inspired hybrid metamaterial with programmable deformation-dependent stiffness and mixed-mode multidirectional auxeticity. The metamaterial can transition from a phase of low stiffness to a contact induced phase that brings forth an extensive rise in stiffness. Uniform and graded configurations of multi-layer tessellated material are developed to modulate the constitutive law of the metastructure with augmented programmability in two- and three-dimensional spaces. The proposed metamaterial will lead to extreme lightweight functional designs for impact resistance, shape morphing, multidirectional deformation, vibration and wave propagation control, where the capabilities of intrinsic material can be most optimally exploited.

## Highlights

• Kirigami-inspired hybrid metamaterial with contact-induced programmable features
• Deformation-dependent stiffness and mixed-mode multidirectional auxeticity
• Uniform and graded configurations of multi-layer tessellated materials
• Extreme lightweight functional designs for most optimal material utilization

## Graphical abstract

**Figure:**
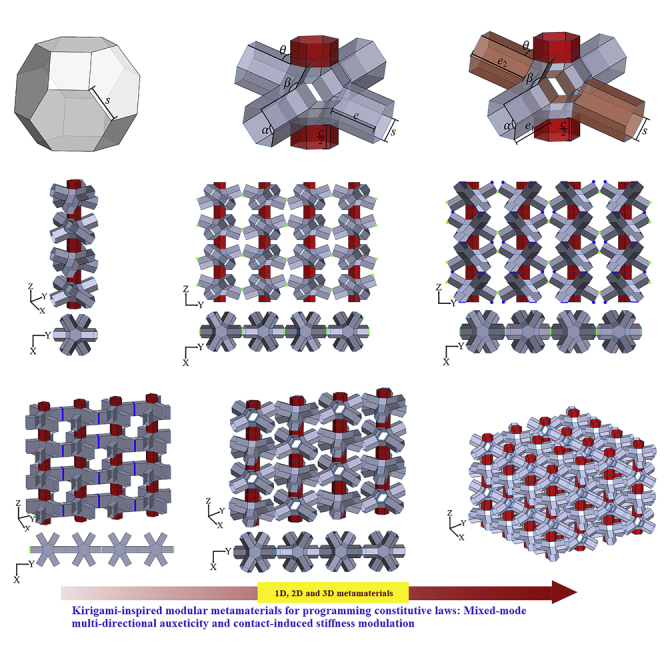

## Introduction

Kirigami, otherwise known as “paper-cutting”, is similar to the better known art of Origami, which involves only folding paper. Kirigami allows for both folding and cutting of a thin sheet to create intricate three-dimensional designs. Kirigami and origami can be seen as models to fabricate deployable structures of a wide spectrum of sizes that may transform their shape without any complicated joints or sophisticated electro-mechanical means (Danesh et al., 2018). A vital benefit of the use of origami and kirigami models from an engineering standpoint is the increased stiffness at the crease, meaning that the increased bending stiffness in the direction of a fold provides a considerable rise in the rigidity of the structures (Dureisseix, 2012). Moreover, the large-scale shape change in origami leads to a range of exploitable mechanical and multi-physics based characteristics with a high compaction ratio. Such tunable characteristics make origami and kirigami based microstructures ideal candidates for architected materials or metamaterials. In a broad sense, these types of materials and structures can be categorized into three classes, origami based systems, kirigami based systems and hybrid origami-kirigami based systems, wherein each class could have rigid facets, deformable facets, and compound rigid-deformable facet behavior (Zhai et al., 2021). In the current article, we would focus on a hybrid origami-kirigami based system with coupled rigid-deformable facet behavior.

With significant potential for a wide range of engineering applications, mechanical metamaterials form a domain of cutting-edge research in the present age (Mukhopadhyay et al., 2020a, 2019a; Christensen et al., 2015; Wu et al., 2021a, 2021b; Isanaka et al., 2022). One of their many defining features is the definition of mechanical properties of the material at a global scale through geometric attributes of the microstructure, rather than only intrinsic material properties of constituent members (Mukhopadhyay and Adhikari, 2016b, 2017b, 2016b; Zschernack et al., 2016; Callens et al., 2019; Meza et al., 2017; Mukhopadhyay et al., 2018; Ghuku and Mukhopadhyay, 2022). This enables meeting specific engineering demands and achieving unusual (not attainable in orthodox natural materials) yet useful mechanical properties, proving beneficial for various multi-functional systems (Zheng et al., 2014; Seepersad et al., 2004; Wang et al., 2020; Singh et al., 2022b).

Recently auxetic metamaterials with negative Poisson’s ratios (Evans et al., 1991; Singh et al., 2022a; Shi et al., 2022), which can be realized through artificial microstructuring (Mukhopadhyay and Adhikari, 2017b; Mukhopadhyay et al., 2020b), are attracting increasing attention because of their enhanced mechanical performances in multiple applications. Over the last two decades, it has been convincingly demonstrated that negative Poisson’s ratio could lead to enhanced impact and indentation resistance (Evans and Alderson, 2000; Alderson et al., 2000; Allen et al., 2015), higher energy absorption (Scarpa et al., 2003; Lakes and Elms, 1993), increased shear stiffness and fracture toughness (Lakes, 1987; Choi and Lakes, 1992), variable permeability, creation of synclastic doubly curved panels, modulation of wave propagation, shape modulation, development of novel actuators and sensors, improved structural designs in terms of stress distribution and deformation control. Different 2D structures have been proposed earlier like re-entrant honeycomb structure (Saxena et al., 2016; Krishnaswamy et al., 2020; Wang et al., 2018; Mukhopadhyay and Adhikari, 2016a), chiral structure (Wojciechowski, 1987,^1989^; Prall and Lakes, 1997), and sheets with perforation and voids (Grima and Gatt, 2010), showing auxetic behavior. Recent advances demonstrate that 3D re-entrant honeycomb structure (Choi and Lakes, 1991), 3D chiral structure (Gao et al., 2018; Fu et al., 2018b), 6-hole Bucklicrystal (Babaee et al., 2013a, 2013b), and 3D double arrow and double ring structures (Yang et al., 2015; Wang et al., 2021) can have auxeticity in three dimensional lattices. Most of these microstructures do not have the ability to show a combination of positive and negative multidirectional Poisson’s ratios in the same microstructure as per functional demands (mixed-modal auxeticity). One of the primary objectives of the current study is to address the aspect of such multi-modal auxeticity, which we propose to realize through adopting kirigami patterns.

For a given origami based metastructural pattern, variation of stiffness with the change in geometric dimensions is well-explored. It has also been shown that a sharp increase in stiffness can be achieved by distorting some origami units (such as forced inversion of vertices) (Waitukaitis et al., 2015; Silverberg et al., 2014). However, such a distortion alters the fundamental behavior of origami or kirigami structures, making them unrecoverable and non-repeatable under cyclic loading. Here we aim to develop a kirigami based material microstructure that has a characteristic ability to seamlessly switch from purely rigid motion to deformable structural behavior under both compressive and tensile loading, resulting in a tunable piece-wise increase in its stiffness and modulation of multidirectional auxeticity.

Kirigami and Origami-inspired metamaterials are receiving increased attention from the scientific community in recent years because of their exciting properties such as space-filling periodicity (Overvelde et al., 2017; He et al., 2020), shape shiftability (Jin et al., 2020), multi-stability (Iniguez-Rabago et al., 2019; Fu et al., 2018a), stretchability (Charkhabi et al., 2019), tunable and graded stiffness (Hwang and Bartlet, 2018; Ma et al., 2018), and auxeticity (Tang and Yin, 2017). Mechanically graded Kirigami materials are being utilized to create robust stretchable electronics (Haque et al., 2021). Artificial cellular structures with polyhedron-based unit-cells have been studied for their ability to support loads at low-density (Yu et al., 2017). Multiple design strategies for reconfigurable materials based on space-filling tessellations of polyhedra have been shown (Overvelde et al., 2017; Iniguez-Rabago et al., 2019; Yu et al., 2017); however, they do not fully exploit the wide range of contact-induced stiffness and auxeticity modulation, and cover a smaller region of the available design space. Thus there exists a strong rationale to exploit the rich capabilities of origami and kirigami based designs for developing novel metamaterials with the constitutive behavior as per application-specific demands for the most effective utilization of the strength and stiffness of a system.

**Figure 1:**
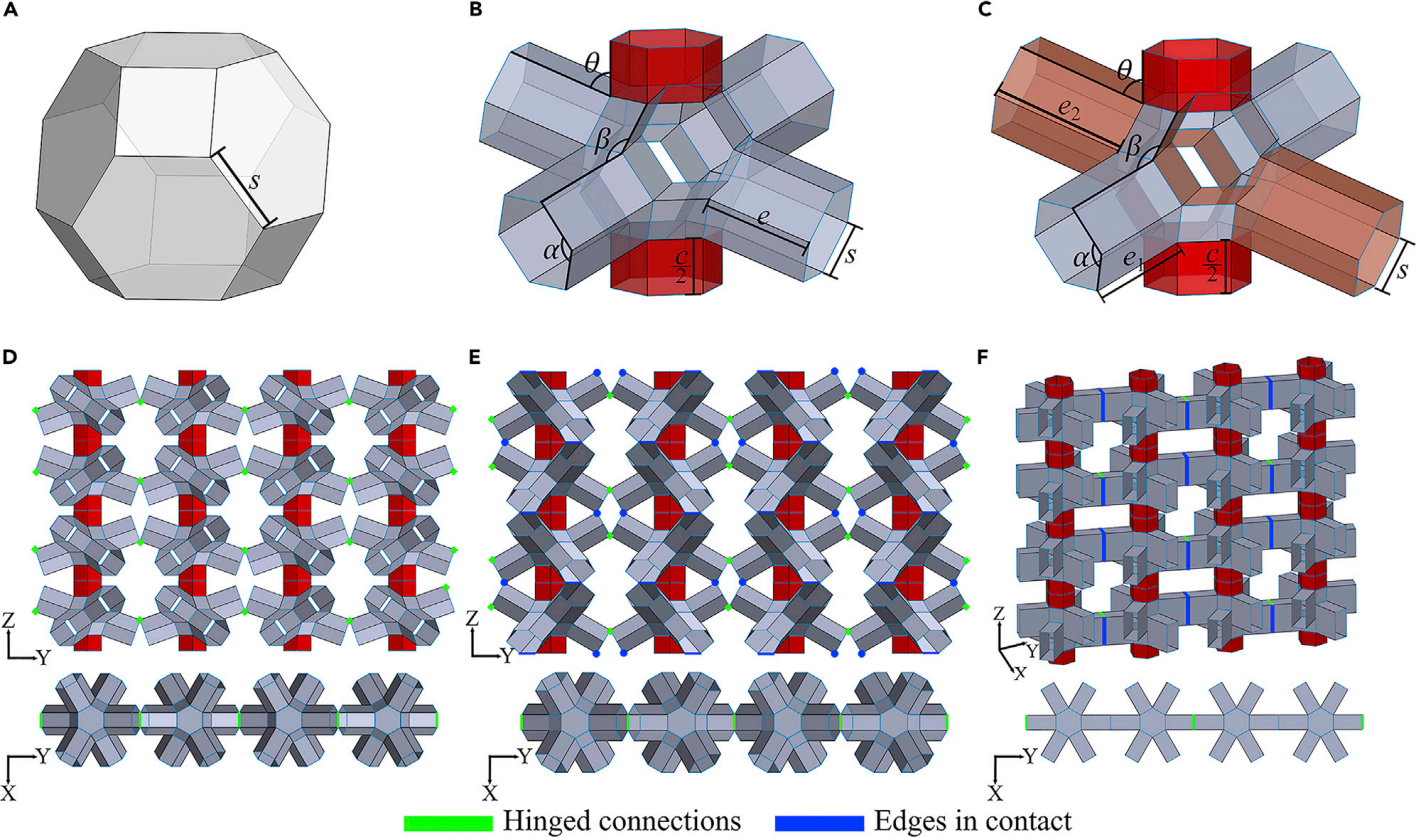
Proposed unit-cells and 2D tessellations for programming constitutive laws (A and B) (A) Typical representation of a truncated octahedron of side length s (B) Geometry of the proposed symmetric unit-cell derived from a truncated octahedron. It can be geometrically defined by a side length (s), extrusion length (e), and connector length (c). (C) Asymmetric unit-cell with in-plane extrusion length $e_{1}$ and out-of-plane extrusion $e_{2}$. Side length (s) and connector length (c) remain the same (note: this configuration is referred to as ‘Asymmetric-I’ later in this paper as we consider another variation of this asymmetric geometry). (D) A two-dimensional tessellation or meta-sheet of the symmetric unit-cell. Unit-cells are attached vertically through their connectors (in red). Horizontally adjacent cells are attached with hinged connections along the edges of their prismatic extrusions (marked in green). A top view of the meta-sheet can be seen at the bottom. (E) Configuration of the meta-sheet on compressive loading. The edges of the prismatic extrusions of vertically adjacent unit-cells that make contact are highlighted in blue. Because the meta-sheet is made through a symmetric unit-cell, both ‘in-plane’ and ‘out-of-plane’ extrusions make contact simultaneously. A top view of this configuration can be seen below. (F) Configuration of the meta-sheet on tensile loading. All corresponding edges of the ‘in-plane’ extrusions of horizontally adjacent unit-cells make contact. All regions of contact are marked by thick blue lines. A top view of this configuration can be seen below.

Motivated by the objectives of programmable modulation of force-deformation constitutive laws and multi-modal Poisson’s ratios, unit-cell (refer to Figure 1B) of the current kirigami metamaterial is proposed based on a truncated octahedron (refer to Figure 1A) with certain faces intuitively replaced by prismatic extrusions. Variation in a pair of these prismatic extrusions further leads to an asymmetric configuration (refer to Figure 1C) that facilitates the bespoke modulation of mechanical attributes of the metastructure. A periodic two-dimensional tessellation of the unit-cell through prismatic connectors (shown in red) and hinges is presented in Figure 1D that would exhibit a steep rise in stiffness during both compressive loading (refer to Figure 1E) and tensile loading (refer to Figure 1F) via contact-induced structural deformation between adjacent unit-cells. The proposed unit-cell would also be able to exhibit in-plane and out-of-plane auxeticity simultaneously, including both auxetic and non-auxetic behavior in a single microstructure. We would demonstrate such multi-functional behavior in the present study by adopting a combined analytical and numerical approach, supported by the elementary-level deformation behavior of physical models.

Firstly, the analytical formulation of the rigid-origami kinematic motion of the proposed unit-cell would be validated through idealized structural simulations. Subsequently, the effects of microstructural geometric parameters on in-plane Poisson’s ratio would be analyzed theoretically. Further, the effects of microstructural geometry would be studied on the constitutive laws of the metamaterial during compressive and tensile loading. Later in this article, the proposed 2D tessellation will be extended to develop a 3D stacked kirigami-based metamaterial with multidirectional auxeticity, which can potentially find critical applications in sensors (Li et al., 2014; Paik et al., 2011; Liu and Hu, 2010), adaptive wings for aircraft (Barbarino et al., 2011; Ha et al., 2016), direction-dependent dynamic behavior, and energy absorption under impact loading (Yu et al., 2017). Essentially we propose to exploit a coupled bilevel design space at the unit-cell level and the lattice level for achieving unprecedented constitutive behavior which can find a wide range of applications.

## Results and discussion

### Microstructural geometric configuration

In this section, first we present detailed geometry of the proposed kirigami metamaterial describing the underlying parameters for modulating mechanical properties of the structural system. Unit-cell of the proposed kirigami metastructure is obtained by taking a uniform truncated octahedron of side length s (shown in Figure 1A) as a template. A truncated octahedron has four pairs of diametrically opposite hexagonal faces. Replacing three of these pairs with prismatic extrusions of length e and adding prismatic extrusions of length c/2 (shown in red) on the remaining pair while keeping other faces rigid, we form the ‘symmetric’ unit-cell shown in Figure 1B. The prismatic extrusion of length c/2 serves as a connector between unit-cells to form tessellations and will hereafter be addressed as a ‘connector’ in this article. Note that although these connectors (i.e., the prismatic extrusion of length c/2) act as links among the unit-cells in the Z direction, we use hinge connections for establishing the links among the unit-cells in the Y direction (as explained later in detail). Thus, a unit-cell is said here to be a truncated octahedron based structure with three pairs of diagonally opposite extrusions and a single pair of diagonally opposite connectors. It can be parameterized by a unique set of s (side length), e (extrusion length), and c (connector length). The reader may note that all lengths and displacements mentioned in this article are of consistent units, and any scale of choice can be adopted for implementation.

An ‘asymmetric’ unit-cell (shown in Figure 1C) is obtained by having two pairs of diametrically opposite extrusions of length $e_{2}$ and the remaining pair of length $e_{1}$ while the connector remains the same. The extrusions of length $e_{1}$ are said to be in-plane whereas the others are out-of-plane. Later in this article, we refer to this asymmetric configuration as the ‘Asymmetric-I’ configuration. The Asymmetric-I unit-cell facilitates gradation in a tessellated structure, which in turn facilitates programmable stiffness. Further variation in the asymmetric configuration leads to the ‘Asymmetric-II’ unit-cell. Here, two of the out-of-plane extrusions have the same length as the in-plane extrusions $\left(e_{1}\right)$, whereas the remaining two out-of-plane extrusions have a length $e_{2}$. The Asymmetric-II unit-cell enables subtle variations in the graded structures for added programmability (refer to contact induced stiffness modulation in method details for further details). Figure 1D shows a tessellation (in the YZ plane) or meta-sheet obtained by forming rigid connections via connectors between vertically adjacent unit cells and by hinged connections between horizontally adjacent in-plane extrusions of respective unit-cells. The hinges facilitate rotation about an edge, and their locations are indicated through bright green markers. Figure 1D also shows the top-view of the meta-sheet, representing a chain of unit-cells attached through hinged connections at the ends of their in-plane extrusions. On providing a sufficient in-plane compressive vertical deformation to the meta-sheet, edges of vertically adjacent extrusions of unit-cells make contact (as depicted by blue markers in Figure 1E), leading to structural deformation and a steep rise in stiffness of the system. Figure 1F represents the physical state of the meta-sheet on a sufficient in-plane tensile vertical deformation for contact and illustrates its top view. Although the green markers represent the hinged edges, blue markers depict edges of in-plane extrusions that make contact, leading to a sharp rise in stiffness during tensile loading. The deformation before contact in both compressive and tensile loading cases is known as rigid-origami or kinematic motion, which depends solely on the torsional rigidity along creases because the panels are assumed rigid at this stage. As shown in Figures 1B and 1C, α is defined as the angle between the highlighted edges of the prismatic extrusions, β as the angle between a square panel and an adjacent prismatic extrusion, and θ as the angle between a prismatic extrusion and a connector. We have established that the kinematics (i.e., pre-contact stage) of the entire structure on deformation can be quantified through any one of these angles, with the others being expressed in terms of it (refer to geometric configuration and kinematic analysis in method details).

### Validation of elementary unit cell kinematics

We study the rigid origami motion of the unit-cell (shown in Figure 1B) by rigidly fixing the six outer nodes (in XY plane) of the bottom connector and applying a longitudinal displacement boundary condition on the outer nodes of the top connector while also restraining their movement in the XY plane. Given a δ vertical deformation to the system, analytical expressions for the values of angles α, β, and θ as a function of δ and the geometric parameter s have been derived as (refer to geometric configuration and kinematic analysis in method details).

(Equation 1) $$\alpha =2\;\sin^{-1}\left(\frac{\sqrt{{\left(\sqrt{6}-\frac{\delta }{s}\right)}^{2}+3}}{2\sqrt{3}}\right)$$

(Equation 2) $$\theta =\sin^{-1}\left(\frac{2\left(\sqrt{6}-\frac{\delta }{s}\right)}{\sqrt{9+3{\left(\sqrt{6}-\frac{\delta }{s}\right)}^{2}}}\right)$$

(Equation 3) $$\beta =\sin^{-1}\left(\frac{{\left(\sqrt{6}-\frac{\delta }{s}\right)}^{2}-3}{\sqrt{9+3{\left(\sqrt{6}-\frac{\delta }{s}\right)}^{2}}}\right)$$

**Figure 2:**
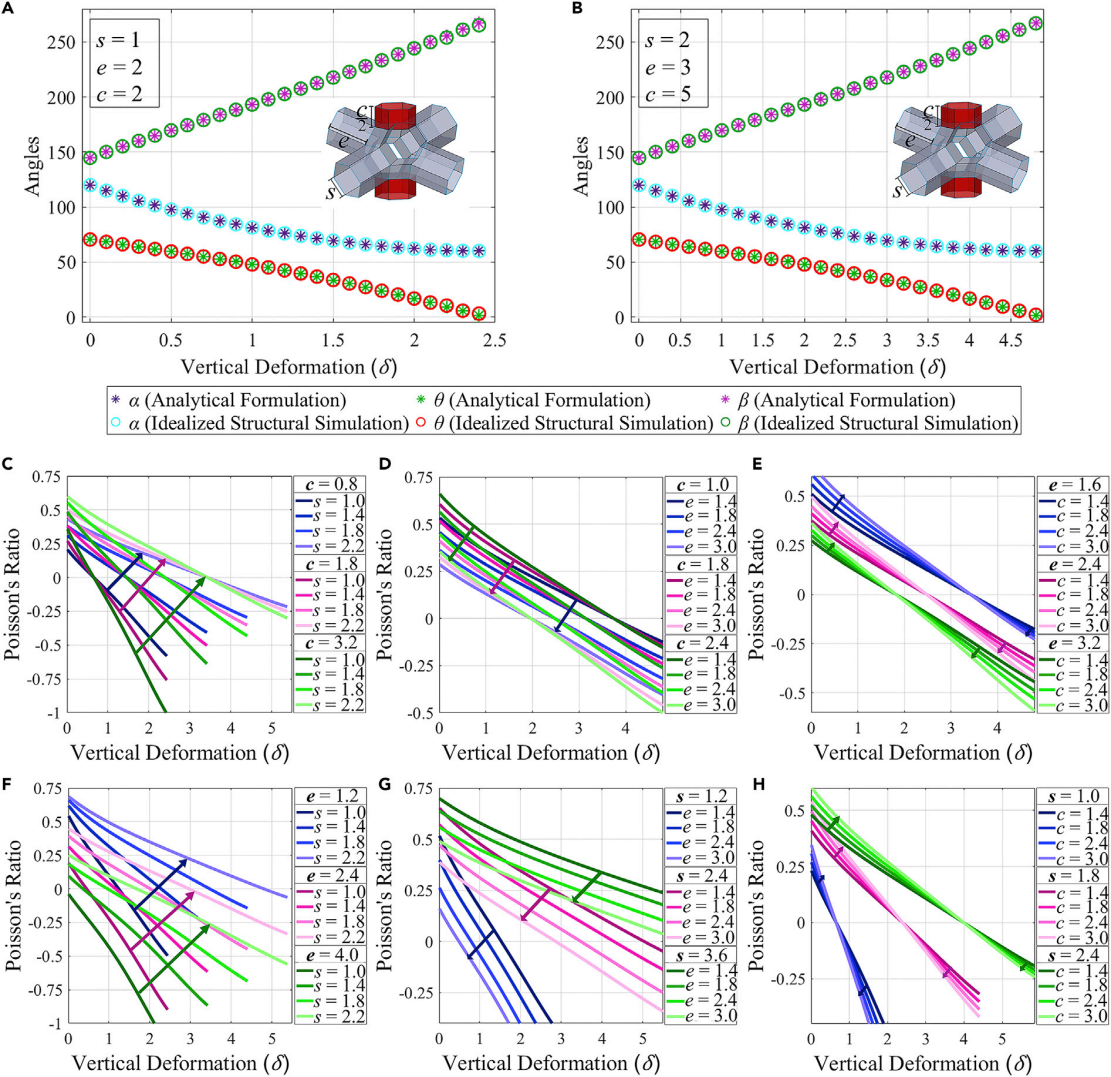
Kinematic behavior and multi-modal Poisson’s ratios of 2D lattices (A) Validation of the magnitudes of angles (α, β and θ) predicted through analytical formulation with results from idealized structural simulations for a range of vertical deformation (δ). Geometrical configuration considered here is s=1, e=2, and c=2. (B) Validation of the magnitudes of angles (α, β and θ) predicted through analytical formulation with results from idealized structural simulations for a range of vertical deformation (δ). Geometrical configuration considered here is s=2, e=3, and c=5. (C) In-plane Poisson’s ratio $\left(\nu_{zy}\right)$ versus Vertical deformation (δ) curves for symmetric unit-cells with constant extrusion length (e=2) and different values of s and c. The arrows depict the trend of higher $\nu_{zy}$ values for greater side lengths (s). (D) In-plane Poisson’s ratio $\left(\nu_{zy}\right)$ versus Vertical deformation (δ) curves for symmetric unit-cells with constant side length (s=1) and different values of e and c. The arrows depict the trend of lower $\nu_{zy}$ values for greater extrusion lengths (e). (E) In-plane Poisson’s ratio $\left(\nu_{zy}\right)$ versus Vertical deformation curves for symmetric unit-cells with constant side length (s=1) and different values of c and e. For positive $\nu_{zy}$, the arrows depict the trend of higher values for greater connector lengths (c)while whereas for negative $\nu_{zy}$, an opposite trend is depicted. (F) In-plane Poisson’s ratio $\left(\nu_{zy}\right)$ versus Vertical deformation (δ) curves for symmetric unit-cells with constant connector length (c=2) and different values of s and e. The arrows depict the trend of higher $\nu_{zy}$ values for greater side lengths (s). (G) In-plane Poisson’s ratio $\left(\nu_{zy}\right)$ versus Vertical deformation (δ) curves for symmetric unit-cells with constant connector length (c=2) and different values of e and s. The arrows depict the trend of lower $\nu_{zy}$ values for greater extrusion lengths (e). (H) In-plane Poisson’s ratio $\left(\nu_{zy}\right)$ versus Vertical deformation (δ) curves for symmetric unit-cells with constant extrusion length (e=2) and different values of c and s. For positive $\nu_{zy}$, the arrows depict the trend of higher values for greater connector lengths (c)whilewhereas for negative $\nu_{zy}$, an opposite trend is depicted.

To validate the proposed analytical expressions (shown in Figures 2A and 2B), we have carried out numerical simulations of the model under longitudinal compression based on an idealized structural analysis with stiffness values described in geometric configuration and kinematic analysis, method details. The idealized structural analysis is performed here using a bar-spring-hinge model (Liu and Paulino, 2016, 2017; Schenk and Guest, 2011) (refer to description of structural analysis for the kirigami based metamaterial in method details for further details). In these simulations, depending on whether the model is undergoing kinematic motion or structural deformation, the extent of structural response is affected by fold stiffness and stiffness of the idealized bar. As the analytical expressions for angles are functions of side length s and vertical deformation δ (refer to Equations 1, 2, and 3), we have considered two unit-cells with different values for side length s each in Figures 2A and 2B. Figures 2A and 2B correspond to unit-cells with s=1 and, respectively. As expected, a higher s increases the overall dimensions of the unit-cell and leads to higher δ requirement for the same α, θ, and β. A good agreement between the results of the numerical and analytical studies presented in Figures 2A and 2B quantitatively substantiates the validity of the subsequent analyses presented in this article, wherein the lattice-level behavior primarily depends on the mechanics of the unit cells. One can further note that the geometrical configurations of the unit-cell at different stages of deformation obtained using idealized structural simulation are in good agreement qualitatively with that of a physical model and a separate full-scale finite element simulation, corroborating considerable confidence in the work (refer to qualitative experiments based on finite element simulations and physical prototypes in method details and Video S1) presented hereafter.

Document S1. Figures S1–S11

### Mixed-mode auxeticity in 2D lattices

Having the unit-cell level deformation behavior validated, we first concentrate on the Poisson’s ratios of 2D lattices that can be obtained by tessellating a unit-cell. Poisson’s ratio can usually be reported through two distinct methods: I. Secant Poisson’s ratio (obtained based on the cumulative deformations between two different points in the deformation path) and II. Tangent Poisson’s ratio (obtained by taking derivative at each instance of the deformation path). One may note that the definition for secant Poisson’s ratio and tangent Poisson’s ratio stated here are alike Poisson’s ratio and Poisson’s function defined in Smith et al. (1999), respectively. Though the main emphasis of this article is on secant Poisson’s ratio, relevant analytical expressions and methods to obtain the tangent Poisson’s ratio for the proposed metastructure are provided in out-of-plane Poisson’s ratio(νzx) of method details for extensiveness. Hereafter in this article, we refer to the secant Poisson’s ratio for all discussions. Usually, for small deformations in conventional materials, the Poisson’s ratio is considered to be constant. However, in the case of large deformation analyses like the current work with Kirigami modular materials, the Poisson’s ratio becomes deformation-dependent. In general, the Poisson’s ratio depicting the deformations along orthogonal directions can be defined as: $\nu_{12}=-\frac{\epsilon_{2}}{\epsilon_{1}}$, where $\epsilon_{1}$ and $\epsilon_{2}$ are strains along the orthogonal directions 1 and 2 (Yasuda and Yang, 2015). Based on this fundamental definition, the in-plane Poisson’s ratio $\nu_{zy}$ for the current kirigami metamaterial is obtained as follows (refer to out-of-plane Poisson’s ratio(νzx) in method details for the detailed derivation).

(Equation 4) $$\nu_{zy}=\frac{\left(\left(\frac{2}{3}\sqrt{9-{\left(\sqrt{6}-\frac{\delta }{s}\right)}^{2}}-\frac{2}{\sqrt{3}}\right)-\frac{4e}{s}\left(\frac{\sqrt{2}}{3}-\frac{\left(\sqrt{6}-\frac{\delta }{s}\right)}{\sqrt{9+3{\left(\sqrt{6}-\frac{\delta }{s}\right)}^{2}}}\right)\right)\left(\frac{6c}{s}+3\sqrt{6}\right)}{\frac{\delta }{s}\left(5\sqrt{3}+\frac{e}{s}4\sqrt{2}\right)}$$

In Equation (73), the Poisson’s ratio is a function of both the microstructural geometry and deformation state (i.e., δ). This is further explored through numerical results presented in Figures 2C–2H. It can be noted in this context that as per the above definition, the Poisson’s ratio is defined for non-zero values of the transverse and longitudinal strains. This implies that Equation (73) will always have non-zero values of δ as input.

By definition, a single unit cell can define the entire periodic metastructure when appropriate boundary conditions are implemented. For example, the Poisson’s ratios of a hexagonal honeycomb lattice can be evaluated by considering only one unit cell as per well-established literature (Gibson and Ashby, 1999; Masters and Evans, 1996). A similar approach has been followed for evaluating Poisson’s ratios in periodic origami based materials (Wang et al., 2020). In the current analysis, a periodic boundary condition is maintained via the hinged connections between horizontally adjacent unit cells. This kind of definition to the Poisson’s ratio and further periodic arraying has been adopted in (Wang et al., 2020). The Poisson’s ratio in Equation (4) represents the entire meta-sheet (valid in the pre-contact regime) because of the nature of the hinged connections made and by the definition of Poisson’s ratio where strains are considered (not deformation). Because the unit cell considered for the analysis here is repeated in a periodic way in the two and three-dimensional spaces, the strain fields are also uniform in a particular direction in such periodic structures. Such uniform strain fields affirm the analogy of analyzing a unit cell for obtaining the global-level properties intuitively.

To study the effects of variation in microstructural geometry on the in-plane (YZ plane) Poisson’s ratio $\nu_{zy}$, in each of the Figures 2C–2H, lengths of two of the three geometric attributes (s, e, and c) are varied whereas the third is kept constant. When kept constant, the side length s has a value of 1.0 whereas extrusion length e and connector length c have a value of 2.0. It can be seen through Figure 2C and 2F that for the same vertical deformation δ, a higher side length s leads to a higher in-plane Poisson’s ratio. A suitable side length can be chosen for the transition from positive to negative Poisson’s ratio after a desirable vertical deformation δ. Consider Figure 2C for example, where a unit-cell with c=3.2 and e=2.0 can be designed for a Poisson’s ratio transition at approximately δ=0.6 or δ=2.4 by setting magnitude of s as 1.0 and 1.8, respectively. Thus, a programmable mixed-mode auxetic behavior can be obtained in a single configuration of the metamaterial. In addition, as shown in the configuration with s=1.0, e=4.0, and c=2.0 in Figure 2F, negative Poisson’s ratio throughout the rigid origami motion phase is also achievable.

Of interest, whenever a variation in connector length c is carried out (Figures 2C–2E and 2H), a reversal in trend is observed after the transition of Poisson’s ratio value from positive to negative. Consider the configurations with s=1.0 and e=2.4 in Figure 2E. For positive values of Poisson’s ratio, a higher c corresponds to a higher Poisson’s ratio, whereas the opposite is true for negative Poisson’s ratio values. An identical deduction can be made from the configurations with s=1.8 and e=2.0 in Figure 2H. Figures 2D and 2G demonstrate that an increase in extrusion length e leads to a decrease in Poisson’s ratio values for the same vertical deformation. One may note that a positive Poisson’s ratio throughout the rigid origami motion phase is also achievable, as shown in the configuration with s=3.6, and c=2.0 in Figure 2G. One can conclude from the results that an increase in s leads to an increase in the Poisson’s ratio for a given δ while whereas the opposite is true for e. Connector length c can be increased for a higher magnitude of Poisson’s ratio, in both positive and negative domains. This confirms that all the three microstructural geometric parameters can be varied for effective Poisson’s ratio modulation. Thus, based on appropriate microstructural configurations, it is possible to obtain a purely auxetic behavior, a purely non-auxetic behavior and mixed-mode auxeticity (i.e., a transition between non-auxetic and auxetic) as per functional demands.

### Programming the constitutive law and stiffness in 2D lattices

**Figure 3:**
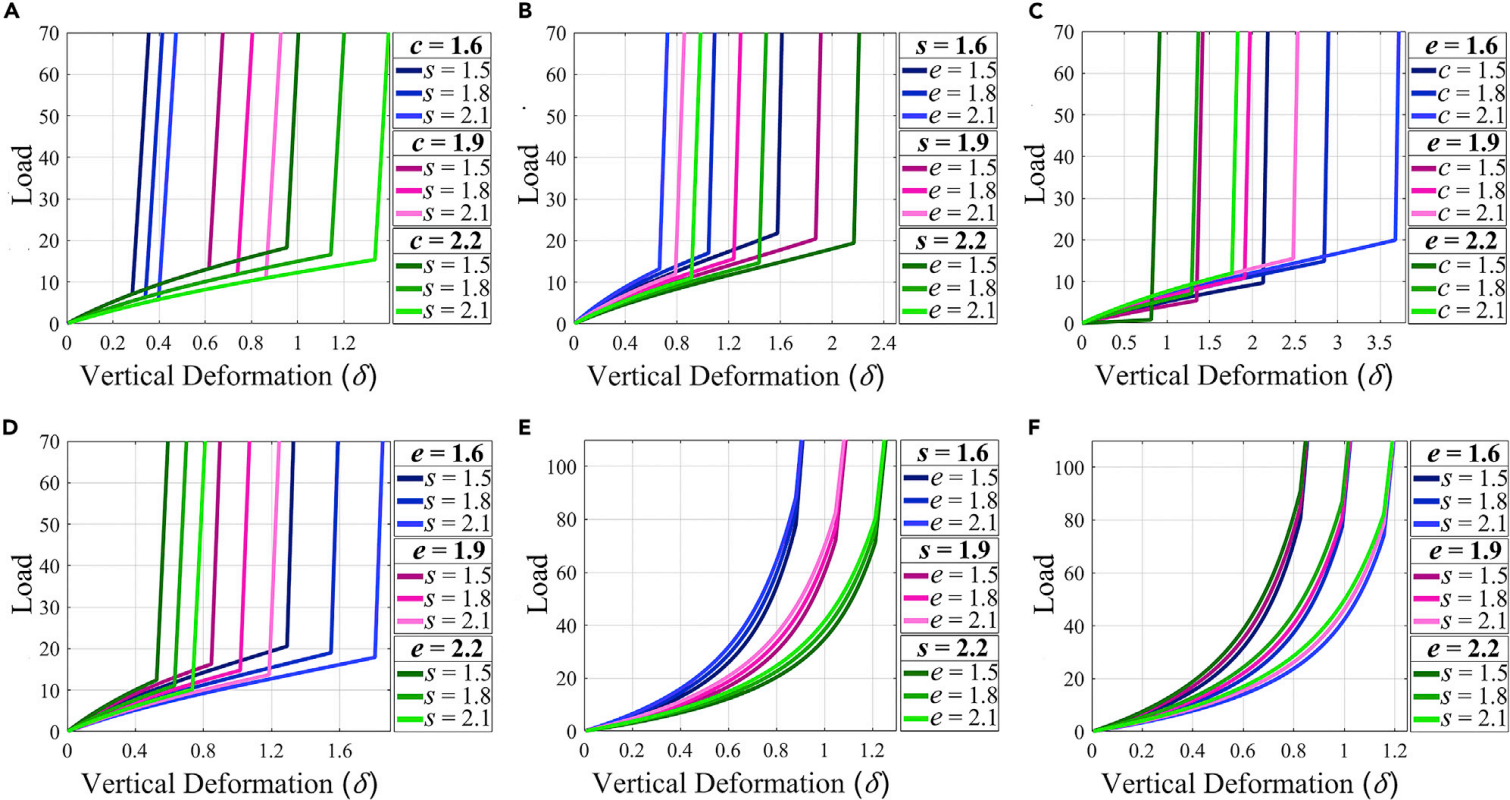
Programming constitutive laws under compression and tension (A) Load versus Vertical deformation (δ) curves for symmetric unit-cells with constant extrusion length (e=2) and different s and c, under compressive loading. Effects of varying s while keeping e and c constant can be seen. (B) Load versus Vertical deformation (δ) curves for symmetric unit-cells with constant connector length (c=2) and different s and e, under compressive loading. Effects of varying e while keeping s and c constant can be seen. (C) Load versus Vertical deformation (δ) curves for symmetric unit-cells with constant side length and different e and c, under compressive loading. Effects of varying c while keeping s and e constant can be seen. (D) Load versus Vertical deformation (δ) curves for symmetric unit-cells with constant connector length (c=2) and different e and cs, under compressive loading. Effects of varying s while keeping c and e constant can be seen. (E) Load versus Vertical deformation (δ) curves for symmetric unit-cells with constant connector length (c=2) and different e and s, under tensile loading. Effects of varying e while keeping c and s constant can be seen. (F) Load versus Vertical deformation (δ) curves for symmetric unit-cells with constant connector length (c=2) and different s and e, under tensile loading. Effects of varying s while keeping c and e constant can be seen. Note here that the loads and displacements plotted here are in consistent units depending on the value of the stiffness properties discussed in description of structural analysis for the kirigami based metamaterial of method details.

To establish a deformation and microstructure-dependent stiffness that can predictively be modeled as a function of the applied far-field deformation, the constitutive relation of the meta-sheet made of symmetric unit-cells under longitudinal load is shown in Figure 3 (by far-field deformation we mean the deformation of a region far away from the applied load boundary condition. In our case, a unit-cell under consideration or point of interest in a sufficiently large domain is assumed to be far away from the loaded edge of the entire meta-sheet, thus experiencing far-field deformation). Note that even though we have presented the results here focusing on the load-deformation behavior in the Z direction, the proposed concepts can readily be extended to explore the constitutive behavior in the Y direction as well. The structural analysis, leading to load-displacement constitutive behavior, is performed with the displacement boundary condition as earlier. Here, the load plotted against vertical deformation is portraying the reaction force in consistent units obtained through the idealized structural simulation. Although Figures 3A–3D demonstrate the constitutive relation for multiple geometric configurations under longitudinal compressive loading, Figures 3E and 3F depict the tensile loading cases. To study the effects of variation in microstructural geometry, in each of the Figures 3A–3F length of two of the three geometric attributes (s, e, and e) are varied whereas the third is kept at a constant length of 2.0. Consider the geometric configuration of s=1.5, e=2.0, and c=2.2 in Figure 3A; a substantial increase in the stiffness during the deformation process is observed when the vertical deformation (δ) given to the system reaches a magnitude of 0.95. Similar transition points marking a steep rise in stiffness are observed in Figures 3A–3F. Of interest, these are the points where pure rigid origami motion ceases and the structural deformation sets in because of contact among the edges of the extrusions of neighboring unit-cells (shown in Figures 1E and 1F). Thus, the behavior of the proposed kirigami metamaterial is not purely rigid but a mixed-mode (rigid and non-rigid) behavior. Initially, under far-field actuation, the metamaterial undergoes a purely rigid deformation (i.e., deformation due to folding along the creases only) until contact among edges occurs. At this point, the facets of the unit-cell must deform to allow any further vertical deformation. Therefore, non-rigid behavior is observed in the post-contact regime. As shown in Equation (84), an expression has been derived (refer to contact induced stiffness modulation in method details for further details) for the compressive loading case to quantify the vertical deformation required for initial contact $\left(\delta_{cc}\right)$ in terms of the geometric attributes of the unit-cell. Similarly an expression for the vertical deformation required for initial contact $\left(\delta_{ct}\right)$ in the case of tensile loading is shown in Equation (85). Therefore, geometric attributes of the unit-cell may be chosen based on when in the deformation process a rise in stiffness is required for a particular application. After the incidence of initial contact, the load can still be transferred through the equivalent bar-hinge-spring system beyond the edges in contact. Thus, afterward (post-contact), a mixed-mode of deformation occurs involving both structural deformation and folding along creases. It can be noted here that the geometrical attributes c and e of the unit-cell must satisfy the inequality depicted in Equation (86) for contact to be possible during vertical deformation (refer to contact induced stiffness modulation in method details for further details).

(Equation 5) $$\delta_{cc}=s\left(\sqrt{6}-3\sqrt{\frac{1-{\left(\frac{c}{2e}\right)}^{2}}{1+3{\left(\frac{c}{2e}\right)}^{2}}}\right)$$

(Equation 6) $$\delta_{ct}=s\left(3-\sqrt{6}\right)$$

(Equation 7) $$\frac{1}{3}<\frac{c}{2e}<1$$

Consider the meta-sheet as shown in Figure 1D; as the length of the connector between vertically adjacent unit-cells is reduced, the prismatic extrusions of the corresponding unit-cells would have a reduced gap between them, implying contact at a lower vertical deformation. The proposed unit-cell is based on a truncated octahedron of side length s, and thus, increasing the side length would imply magnifying the base structure of the unit-cell while keeping the prismatic extrusions of constant length. This would, in turn, magnify the gap between adjacent prismatic extrusions too and cause contact at a higher vertical deformation. Consider Figure 3A; values of $\delta_{cc}$ for configurations with e=2.0 and c=1.9 can be compared to confirm their rise in magnitude with an increase in s. Configurations with constant c in Figure 3D also confirm the relation between magnitudes of s and $\delta_{cc}$. In Figure 3C, configurations with s=2.0 and e=1.6 depict the rise in $\delta_{cc}$due to larger magnitudes of c. One can observe that as the lengths of the prismatic extrusions are increased, the gap between vertically adjacent extrusions is reduced, leading to initial contact at a lower vertical deformation. In Figure 3B, as the extrusion length is increased for a fixed value of s and c, initial contact occurs at a smaller vertical deformation. The same can be confirmed by observing values of $\delta_{cc}$ for configurations with c=2.0 and s=1.9. It can also be noted that for the same e, the sudden increase in stiffness occurs at a higher vertical deformation for a larger value of s, as predicted earlier. Another important takeaway from Figure 3D is that, for an identical rise in s, a higher e corresponds to a larger rise in $\delta_{cc}$. A similar analogy can be formed between s and c by observing the trends in Figure 3A. Thus, it can be pointed out that the magnitude of $\delta_{cc}$ can be increased (via rise in s and c) and decreased (via rise in e), establishing the stiffness modulation properties of the metamaterial. One may note that the stiffness in the post-contact region is roughly the same for all configurations because the exact same set of edges come into contact, as can be observed from the meta-sheet in the post-contact regime shown in Figure 1E. However, the curvature and slopes of each of the two legs of the constitutive curves depend on the respective microstructural parameters and mode of deformation (i.e., tensile and compressive).

Consider the meta-sheet under tensile loading as shown in Figure 1F. Here contact takes place between horizontally adjacent in-plane extrusions as compared to contact between vertically adjacent extrusions in the compressive loading case. Comparing Figures 1E and 1F, it can be seen that although both out-of-plane and in-plane extrusions make contact in the compressive loading case, only in-plane extrusions make contact in the tensile loading case. As explained earlier, two horizontally adjacent unit cells are attached through a hinged connection along one of the outer edges of their extrusions. As depicted in Figure 1F, the remaining five edges of an in-plane extrusion make contact with corresponding edges of the adjacent prismatic extrusion. Because this contact occurs in a horizontal fashion (as opposed to the vertical fashion in compression), the critical point of initial contact is not dependent on the length of the prismatic connector. As described earlier, increasing the side length s while keeping the extrusion length e constant would magnify the gap between horizontally adjacent prismatic extrusions. It can be seen in Figures 3E and 3F that an increase in side length s correlates with contact occurrence at a higher vertical deformation. Unlike the trends seen in compression loading, Figure 3F shows that, for an identical rise in s, a higher e does not correspond to a larger rise in $\delta_{ct}$. This is because of the fact that $\delta_{ct}$ is only a function of s (refer to Equation (85)).

It becomes clear from the discussions of the preceding paragraphs that the steep rise in stiffness of the metamaterial can be programmed as a function of deformation (or applied end-force of tensile and compressive mode) according to the explicit requirement of a system or structure. It can further be noted that the proposed metamaterial is capable of exhibiting non-invariant force-deformation constitutive behavior under tensile and compressive mode of loading, which is contrary to the common mechanical behavior of conventional naturally occurring materials. In this context, it may be noted that such programmable large deformation constitutive modeling is normally not possible in lattice metamaterials.

A graded microstructure is often implemented in metamaterials to achieve variable stiffness in the system or structure as to where it is needed (i.e., spatial variation of stiffness). Through the proposed metamaterial, we demonstrate that it is also possible to accomplish variable stiffness as to when in the deformation process it is desired (i.e., temporal variation of stiffness). Thus, we can now propose meta-sheets developed with unit-cells of different geometrical attributes to vary stiffness as to where in the structure and as to when in the deformation process it is needed. One can observe that to make a meta-sheet as proposed in Figure 1D, the in-plane features of the unit-cell must be uniform; else, the prismatic extrusions may not be aligned for hinged connections. Consecutive unit-cells in a single column would also be required to have the same side-length s for the possibility of attachment through their hexagonal connectors. Certain variations in connector length are possible. However, we have restricted this study to variations in out-of-plane extrusions in a meta-sheet. As shown in Figures 1B and 1C, ‘asymmetric-I’ unit-cells are defined by out-of-plane extrusions of different lengths, compared to the in-plane extrusions (note that we refer to this configuration as ‘asymmetric-I’ because another variation of asymmetric unit-cell is proposed in the following paragraphs).

In the case of compressive loading for symmetric unit-cells (Figures 3A–3D), both the in-plane and out-of-plane extrusions make contact at an equal amount of vertical deformation. However, in the case of ‘asymmetric-I’ unit-cells, depending on whether the out-of-plane extrusions are longer or shorter than the in-plane extrusions, they will make contact earlier or later, respectively. This dual contact behavior in an ‘asymmetric-I’ unit-cell will lead to two ‘jumps’ in stiffness as compared to the single jump in stiffness of symmetrical unit-cells (refer to contact induced stiffness modulation in method details for details). In this study, we have considered asymmetric-I unit-cells with larger values of $e_{2}$ as compared to $e_{1}$ to obtain sets of unit-cells for the graded microstructural geometry.

**Figure 4:**
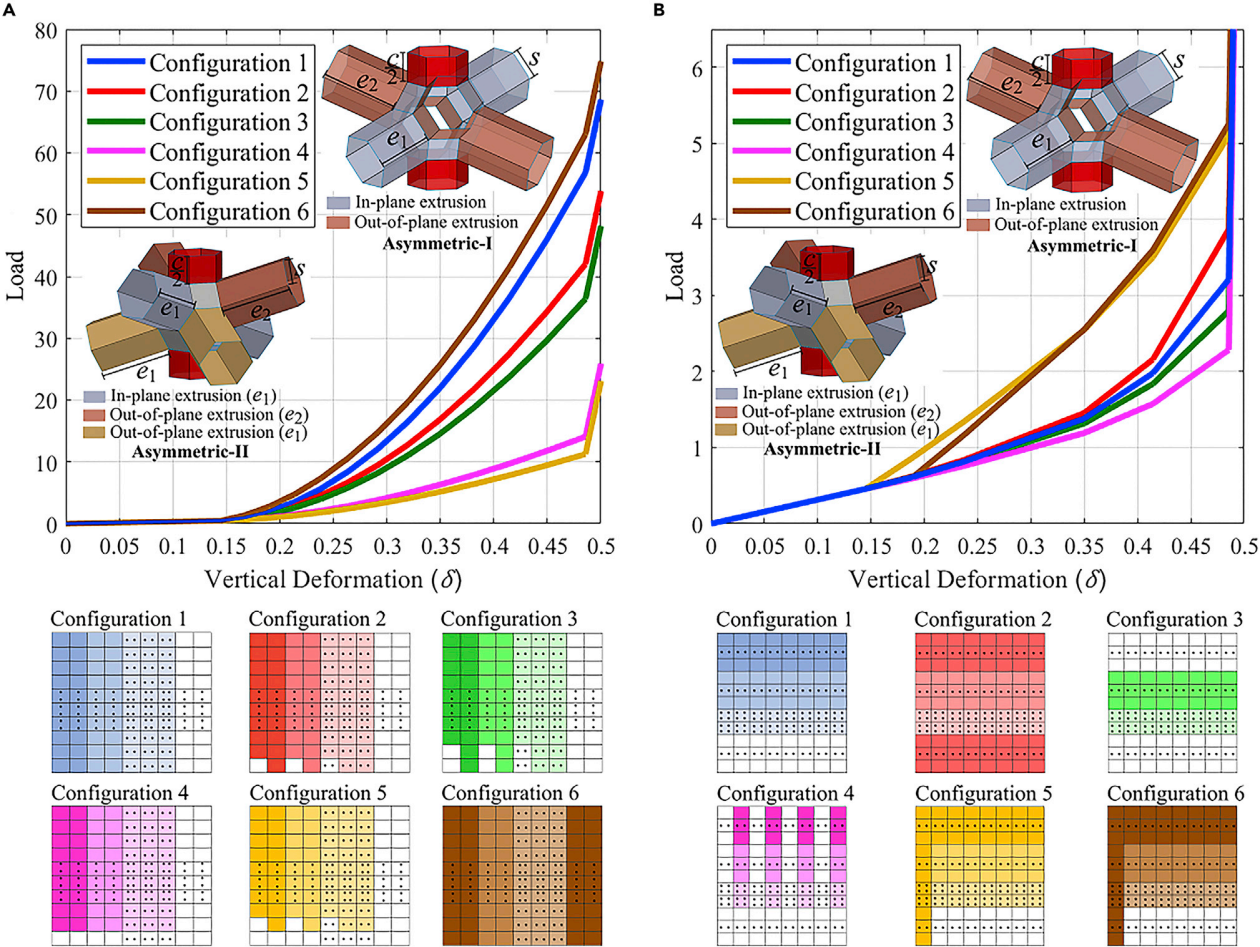
Piece-wise constitutive law modulation under compression for 2D kirigami lattices with asymmetric unit-cells (A) Load versus Vertical deformation (δ) curves for meta-sheet with ‘row-wise’ gradation. $e_{2}$ is varied from a magnitude of 2.6 to 2.0 with decrements of 0.05 to create a set of 13 unit-cells. The side length (s) of all unit-cells is 1.0 whereas the connector length (c) and ‘in-plane’ extrusion length $e_{1}$ are fixed as 2.0. Each row of Configuration-1 consists of two ‘asymmetric-I’ unit-cells of each type, and each column consists of 26 unit-cells of a kind, in turn forming a grid size of 26×26. Configurations are represented by a color gradient correlating to the decreasing $e_{2}$ along a row. All other configurations are based on Configuration-1, with systematic changes in the lattice to achieve varied stiffness. Configuration-2 has the bottom-most unit-cell of odd columns replaced by a symmetric unit cell. Configuration-3 has the two bottom-most unit-cells of odd columns replaced by symmetric unit-cells. Configuration-4 has the bottom-most unit-cell of every column replaced by a symmetric unit cell. Configuration-5 and Configuration-6 are formed by replacing a column of the original meta-sheet with a column consisting completely of ‘asymmetric-I’ unit-cells with $e_{2}$ of 2.6 and 2.5, respectively. Because all 26 unit cells cannot be shown in the grids, square cells containing dots are added to represent the intermediate unit cells, columns or rows. (B) Load versus Vertical deformation (δ) plots for meta-sheet with ‘Column-wise’ gradation. $e_{2}$ is varied from a magnitude of 2.6 to 2.0 with decrements of 0.1 to create seven unit-cells. Each $e_{2}$ corresponds to a ‘set’ of four consecutive unit-cells attached through connectors. The first and fourth unit-cells are ‘asymmetric-II’ whereas the remaining are ‘asymmetric-I’ unit-cells. The side length (s) of all unit-cells is 1.0 whereas the connector length (c) and ‘in-plane’ extrusion length $e_{1}$ are fixed as 2.0. Each column of Configuration-1 consists of 7 ‘sets’ and each row consists of 28 unit-cells of a kind, in turn forming a grid size of 28×28. Configurations are represented by a color gradient correlating to the decreasing $e_{2}$ along a column. All other configurations are based on Configuration-1, with systematic changes in the lattice to achieve varied stiffness. Configuration-2 has the bottom-most ‘set’ replaced by a ‘set’ corresponding to an $e_{2}$ of 2.6. Configuration-3 has the top most ‘set’ replaced by a ‘set’ corresponding to an $e_{2}$ of 2.0. Configuration-4 is formed by replacing half (alternate) of the columns with columns of symmetric unit cells. Configuration-5 and Configuration-6 are formed by replacing a single column with a column consisting of ‘asymmetric-I’ unit-cells with $e_{2}$ of 2.6 and 2.5, respectively. Because all 26 unit cells cannot be shown in the grids, square cells containing dots are added to represent the intermediate unit cells, columns or rows. In these plots, we consider a displacement-controlled analysis with the assumption of two parallel edges during any stage of deformation, whereas the vertical displacement is applied perpendicular to the horizontal edges. Note here that the loads and displacements plotted here are in consistent units depending on the value of the stiffness properties discussed in description of structural analysis for the kirigami based metamaterial in method details.

We have proposed two kinds of graded meta-sheets based on the direction of gradation. These meta-sheets can be designed with any numerical configuration of unit cells. However, for this study, we have considered configurations based on the ease of graphical representation and to portray the interesting trends. In Figures 4A and 4B, we have provided constitutive laws for the complete meta-sheets, calculated through the stiffness derived from constitutive laws of individual unit cells. The assembly of unit cells in a meta-sheet is analogous to a lattice network made from mechanical springs in series and parallel connections. Thus, the equivalent stiffness of the spring network based on the occurrence of sequential contact is followed in obtaining the load versus vertical deformation (δ) behavior. One can note that because the in-plane geometrical attributes are fixed in this study, the proposed meta-sheets would have a proper edge-to-edge contact even in the case of tensile loading. It can further be noted that we provide displacement boundary conditions at the edges, meaning that two opposite edges parallel to each other before deformation remains parallel after deformation.

Earlier, we have shown that in compressive loading, edges of the extrusions of vertically adjacent unit-cells make contact. For proper edge-to-edge contact between extrusions pointing toward each other (for two vertically adjacent unit-cells), their lengths $\left(e_{2}\right)$ must be the same. This is the case because the vertical deformation required for initial contact is a function of $e_{2}$, as shown in Equation (84). This poses a problem in cases where two ‘asymmetric-I’ unit-cells of different $e_{2}$ magnitudes are attached vertically. To satisfy this condition, we have introduced the ‘asymmetric-II’ unit-cell, as shown in Figure 4 (refer to contact induced stiffness modulation in method details for further details). One can observe that out of the four out-of-plane extrusions (in brown) of the ‘asymmetric-I’ unit-cell (shown in Figure 4), two point in the upward direction whereas two point in the downward direction. An ‘asymmetric-II’ unit-cell has one of these pairs of extrusions (in yellow) of length equal to that of in-plane extrusions $\left(e_{1}\right)$ of the meta-sheet whereas the other pair (in brown) of a different length $e_{2}$. Thus, ‘asymmetric-II’ unit-cells can be used at interfaces of two different asymmetrical unit-cell groups. For example, consider a column consisting of an even number of ‘asymmetric-I’ unit-cells. If the top and bottom half consist of ‘asymmetric-I’ unit-cells of different $e_{2}$, a pair of ‘asymmetric-II’ unit-cells can replace the ‘asymmetric-I’ unit-cells at the interface, enabling proper contact. The unit-cell above the interface and the unit-cell below the interface would have their downward-pointing and upward-pointing extrusions of the same length $\left(e_{1}\right)$, respectively (refer to contact induced stiffness modulation in method details for further details).

Figure 4A demonstrates a ‘row-wise’ graded meta-sheet where gradation is seen along a row. As explained earlier, the longer the out-of-plane extrusions, the earlier they make contact during the vertical deformation process. Here gradation is defined as a gradual decrease in lengths of out-of-plane extrusions of unit-cells in a left-to-right direction along a row. Thus, successive unit-cells from left to right would make initial contact as vertical deformation proceeds. However, because the in-plane extrusion length $e_{1}$ is uniform, all unit-cells would make the second (in-plane) contact at the same vertical deformation. Here, the $e_{2}$ of successive pairs of unit-cells in a row is varied from a magnitude of 2.6 to 2.0 with decrements of 0.05 to create a group of 13 unit-cells. For all unit-cells, connector length (c) and side length (s) are fixed as 2.0 and 1.0 respectively. The in-plane parameter $e_{1}$ of all unit-cells is fixed as 2.0. Thus, the rightmost pair of unit-cells in a row are symmetric unit-cells with, e=2.0, c=2.0 (both in-plane and out-of-plane extrusions of same length). Therefore, each row contains 26 unit-cells, and we have attached 26 of these rows as depicted earlier in Figure 1D, leading to a meta-sheet containing 26×26 unit-cells. Although a myriad of configurations of the meta-sheet is possible, here we present six configurations, each based on the row-gradation as explained before but with subtle alterations leading to unique constitutive laws. Configurations are represented through grids of unit-cells and a color gradient relating to the decreasing magnitude of $e_{2}$ along a row.

The stiffness curve can be divided into three broad sections defined by the first and final contacts in the meta-sheet. Using Equation (84), we can find that unit-cells containing out-of-plane extrusions of length 2.6 and 2.0 make the first contact at vertical deformations of 0.1448 and 0.4855 respectively. The first section is defined until the first contact; here, the meta-sheet undergoes rigid-origami motion leading to a comparatively minute value of stiffness. In Figures 4A and4A gradual increase in stiffness can be observed after a vertical deformation of 0.1448 until a vertical deformation of 0.4855. This is the second section defined by a steady rise in stiffness through successive contacts between out-of-plane extrusions as deformation progress. Henceforth is the third section where all extrusions of the meta-sheet are under contact. A steep rise in stiffness similar to that observed in Figure 3 can be seen here.

Each row of Configuration-1 consists of pairs of the 13 unit cells arranged as described earlier, and each column consists of 26 unit cells of a kind. Because all 26 unit cells cannot be shown in the grids, square cells containing dots are added to represent the intermediate unit cells, columns or rows. In accordance with the color gradient scheme, the penultimate and final symmetric unit cells at the end of each row are represented by a white square cell. A slight decrease in stiffness in the second section is shown by replacing either the final unit-cell or both the final and penultimate unit-cells of odd columns by a symmetric unit-cell in configurations 2 and 3, respectively. By replacing asymmetrical unit-cells with symmetric unit-cells, contribution to the net stiffness in the second section decreases because contact happens only at the very end of the section. As expected, a steep drop in stiffness in the second section is seen when the final unit-cell of every column is replaced by a symmetric unit-cell in Configuration-4. Further decrease in stiffness can be seen with Configuration-5, where the final unit-cell of every column along with the second-last unit-cell of odd columns are replaced by symmetric unit-cells. The ‘asymmetric-I’ unit-cells present at the created interface are replaced by ‘asymmetric-II’ unit-cells in these configurations. One way of increasing the stiffness in the second section is by replacing the two columns of symmetric unit-cells in Configuration-1 with columns of asymmetrical unit-cells with an $e_{2}=2.6$, forming Configuration-6. This causes an added increase in stiffness at the beginning of the second section, as depicted by the brown curve in Figure 4A. One should note that modulation in net stiffness of the meta-sheet can also be brought about by changing the number of unit cells in the columns or rows.

In Figure 4B, we have proposed a ‘column-wise’ gradation for comparatively lower magnitudes and fine modulation of stiffness. Here, a gradation in the out-of-plane extrusion lengths of unit-cells is seen along a single column of the meta-sheet. We have proposed dividing a column into multiple ‘sets’ with each set consisting of consecutive unit-cells of the same geometry. The gradation is achieved through changes in these ‘sets’ along a column with each consecutive set consisting of unit-cells with smaller $e_{2}$ magnitudes as compared to the previous set. As shown in configuration-1 of Figure 4B, all unit-cells in a ‘set’ are represented by the same color, and a color gradient is shown to depict the decreasing length of out-of-plane extrusions as we move from top to bottom in a column. Thus, successive ‘sets’ from top to bottom would make initial contact as vertical deformation proceeds. However, because the in-plane extrusion length $e_{1}$ is uniform, all unit-cells would make the second contact at the same vertical deformation.

One can note that at the interface of two different sets in a column, two different unit-cells would be making contact (refer to contact induced stiffness modulation in method details for further details). ‘Asymmetric-II’ unit-cells are used here, which have one pair of out-of-plane extrusions of length $e_{1}$while whereas the other pair of a length equal to the $e_{2}$ magnitude of the set it belongs to. Thus, at an interface of ‘sets’ the unit-cell above the interface and the unit-cell below the interface would have their downward-pointing and upward-pointing extrusions of the same length $\left(e_{1}\right)$, respectively. A set of ‘n’ members would therefore consist of two ‘asymmetric-II’ unit-cells at the ends and n−2 ‘asymmetric-I’ unit-cells in between. Note that the ‘asymmetric-I’ and ‘asymmetric-II’ unit-cells are represented similarly in the grids of different configurations proposed. Here, each ‘set’ consists of 4 unit-cells, and $e_{2}$ of successive sets in a column is varied from a magnitude of 2.6 to 2.0 with decrements of 0.1 to create a total of 7 sets. For all unit-cells, connector length (c) and side length (s) are fixed as 2.0 and 1.0 respectively. The in-plane parameter $e_{1}$ of all unit-cells is fixed as 2.0. Thus, the bottom-most set in a column consists of symmetric unit-cells with s=1.0, e=2.0, c=2.0. Each column contains 28 unit-cells, and we have attached 28 of these columns through hinges as depicted in Figure 1D, leading to a meta-sheet containing 28×28 unit-cells. The six configurations presented in Figure 4B are based on the column-gradation as explained above but with certain alterations leading to fine modulation in constitutive laws.

Configurations are represented by a color gradient correlating to the decreasing magnitude of $e_{2}$ along a column. The ‘asymmetric-I’ unit-cells in each set are represented by a square cell with two dots, whereas the complete set is depicted in the same color. For brevity, the third to sixth sets of each column in the configurations are represented by squares containing four dots. Similar to the ‘row-wise’ configuration, the stiffness curves can be divided into three broad sections defined by the first and final contacts in the meta-sheet. As defined earlier, unit-cells with out-of-plane extrusions of length 2.6 and 2.0 make the first contact at vertical deformations of 0.1448 and 0.4855 respectively. As shown in Figure 4B, changes in the stiffness curves can be induced in the second section (between a vertical deformation of 0.1448 and 0.4855) by subtle variations in the meta-sheet.

Each column of Configuration-1 consists of 7 sets of 4 unit-cells arranged as described earlier, and each row consists of 28 unit-cells of a kind. A slight increase in stiffness is shown by replacing the seventh set of symmetric unit-cells with the first set corresponding to an $e_{2}$ of 2.6 in Configuration-2. Similarly, a decrease in stiffness can be achieved by replacing the first set of unit-cells with the seventh set of symmetric unit-cells as shown by Configuration-3. A greater decrease in stiffness can be achieved by replacing half (alternate) of the columns in the original meta-sheet with columns of symmetric unit-cells as demonstrated in Configuration-4. Although Configurations 1–4 have demonstrated a gradual increase in stiffness, it is also possible to facilitate a jump in stiffness in the second section. Configuration-5 and Configuration-6 are formed by replacing a column of the original meta-sheet with a column consisting of ‘asymmetric-I’ unit-cells with $e_{2}$ of 2.6 and 2.5, respectively. Corresponding to an $e_{2}$ of 2.6 and 2.5, jumps in stiffness can be seen at a vertical deformation of 0.1448 and 0.1894. Thus, stiffness jumps can be programmed at required vertical deformations by using relevant configurations. The extreme rise in stiffness in the third section is achieved by contact of all in-plane extrusions as earlier explained for ‘row-wise’ configurations.

As discussed in this section, by adopting graded unit-cells (i.e., spatially varying asymmetric unit-cells) in the 2D tessellation (column and row-wise), it is possible to obtain piece-wise programming of stiffness (both increase and decrease in the rate of change, as per functional demands) and force-displacement constitutive behavior with an increased scope of modulation.

### 3D programmable hybrid tessellations

**Figure 5:**
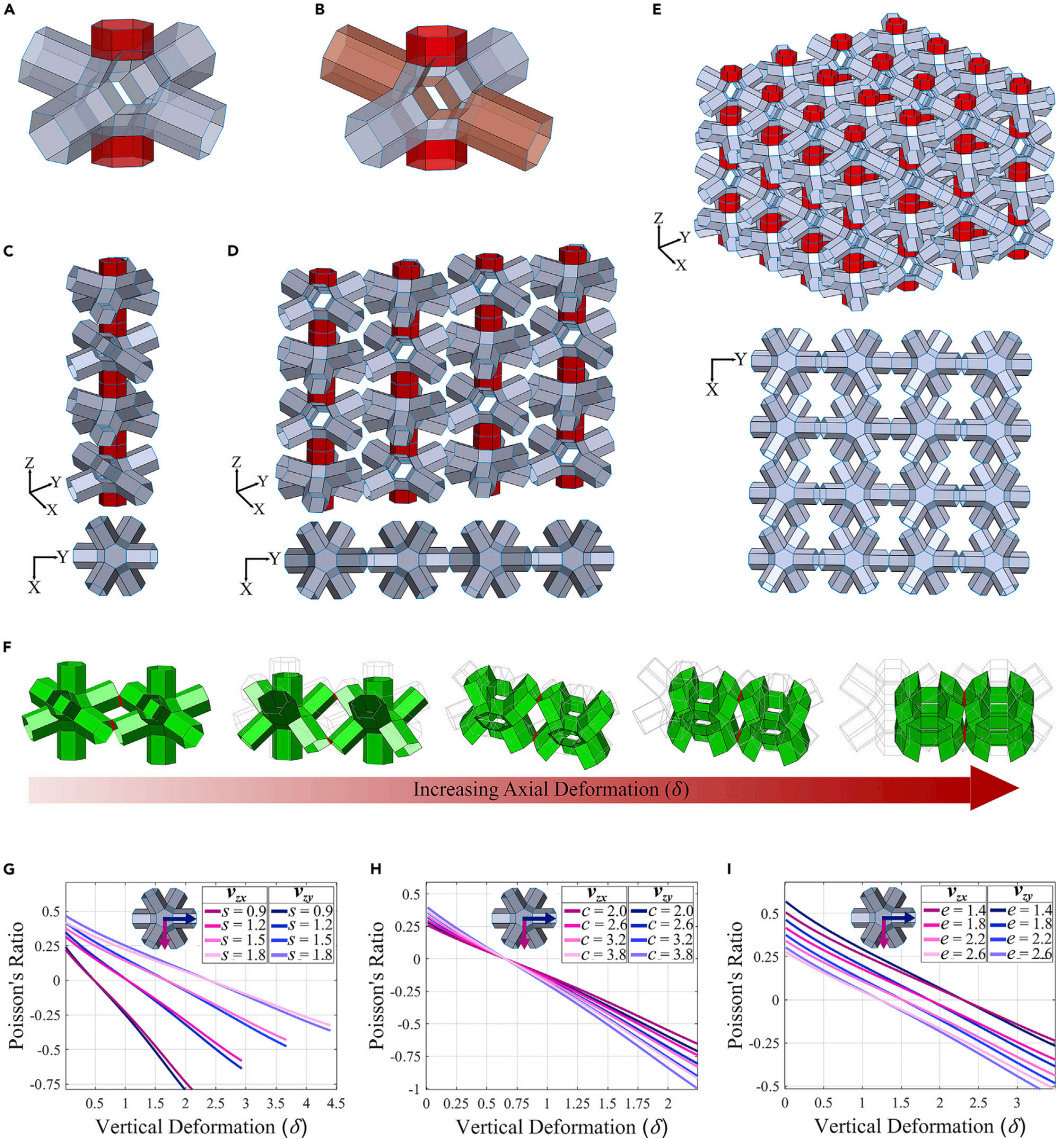
3D tessellation and the concept of multidirectional auxeticity modulation (A and B) Symmetric unit-cell. (B) Asymmetric-I unit-cell. (C) Single column of 4 symmetric unit-cells attached through connectors. The top view is presented below. (D) 2D meta-sheet of 16 symmetric unit-cells attached through connectors and hinges between horizontally adjacent unit-cells. The top view is presented below. (E) 3D meta-structure of 64 symmetric unit-cells attached through connectors and hinges between laterally adjacent unit-cells. The metastructure is obtained through hinged connections (similar to that in 2D meta-sheets) between the out-of-plane extrusions of meta-sheets. The top view is presented below. (F) Idealized structural simulation of a pair of unit-cells attached through an out-of-plane hinged connection (in red). Kinematic motion can be observed as axial deformation (δ) is increased. The initial undeformed state of the unit-cell pair is indicated via a thin outline. (G) Poisson’s ratio versus vertical deformation (δ) comparison between out-of-plane $\left(\nu_{zx}\right)$ and in-plane $\left(\nu_{zy}\right)$ cases. Side length (s) is varied whereas e=2.0 and c=2.0. (H) Poisson’s ratio versus vertical deformation (δ) comparison between out-of-plane $\left(\nu_{zx}\right)$ case and in-plane $\left(\nu_{zy}\right)$ case. Connector length (c) is varied whereas s=1.0 and e=2.0. (I) Poisson’s ratio versus vertical deformation (δ) comparison between out-of-plane $\left(\nu_{zx}\right)$ case and in-plane $\left(\nu_{zy}\right)$ case. Extrusion length (e) is varied whereas s=1.0 and c=2.0.

In this section, we present the strategies to extend the proposed 2D hybrid origami-kirigami tessellation to 3D domain. Figure 5 illustrates the process of forming a three-dimensional metamaterial from a single symmetric unit cell. Figures 5A and 5B depict the symmetric and ‘asymmetric-I’ unit-cells, respectively. Rigidly attaching unit cells in a longitudinal fashion through the connector extrusions shown in red leads to a single column in Figure 5C. As expected, the top view of the column is that of a single unit cell because of the chosen arrangement. Figure 5D gives an isometric representation of the 2D meta-sheet (in the YZ plane) with its top orthogonal view. As discussed earlier, adjacent columns are attached via hinges that facilitate rotation about their axes. Although the meta-sheet is formed via connections between in-plane extrusions of columns, the 3D tessellation is obtained through similar hinged connections between the out-of-plane extrusions of meta-sheets.

The particular hinged edges of out-of-plane extrusions can be observed in the orthogonal top view of the 3D tessellation shown in Figure 5E. The isometric view of a 4×4×4 three-dimensional tessellation formed in this manner can also be seen in Figure 5E. An idealized structural simulation is performed to observe motion behavior for a pair of unit-cells with hinged connections through their out-of-plane extrusions. Figure 5F shows the geometric state of the unit-cell pair as vertical deformation (δ) is increased, with the hinged edges highlighted in red. Although the bottom vertices of the right unit-cell are rigidly fixed, the bottom vertices of the left unit-cell are restrained from any out-of-plane displacement only. A uniform compressive displacement boundary condition is applied to the top vertices of both the unit-cell. As vertical displacement is increased, one can observe that the unit-cell pair tends to move toward each other, displaying a prospective auxetic behavior for the 3D tessellation formed using such unit-cell connections. The initial undeformed geometry of the unit cells can be seen as a faint gray outline. Thus, from this, one can understand how the three-dimensional tessellation shown in Figure 5E would behave under compressive loading.

Previously in Figure 2, we had discussed the programmable in-plane (YZ plane) Poisson’s ratio of the unit-cell. By stacking meta-sheets in the out-of-plane direction (along the Xaxis), we can now introduce the out-of-plane (XZ plane) Poisson’s ratio represented as $\nu_{zx}$ according to earlier notation. As shown in Equation (81), the Poisson’s ratio is a function of both the microstructural geometry and deformation state (refer to out-of-plane Poisson’s ratio(νzx) in method details for detailed derivation). Figures 5G–5I depict a comparison between the in-plane $\left(\nu_{zy}\right)$ and out-of-plane $\left(\nu_{zx}\right)$ Poisson’s ratios by varying the microstructural attributes. The top view of a unit-cell is also shown in the insets with in-plane and out-of-plane directions clearly marked. A symmetric unit-cell is considered with a base configuration of s=1.0, c=2.0, and e=2.0. Figures 5G–5I vary s, c, and e respectively while keeping the remaining two geometrical attributes of base configuration length. In each of Figures 5G–5I, it can be observed that both Poisson’s ratios have very close magnitudes and follow the same trends. However, a subtle difference is that the in-plane Poisson’s ratio has a higher magnitude for negative values whereas the opposite is true for positive values. Note that a deformation-dependent programmable transition of Poisson’s ratios between the auxetic and non-auxetic modes can be achieved for both the in-plane and out-of-plane directions.

(Equation 8) $$\nu_{zx}=\frac{\left(\left(\sqrt{9-{\left(\sqrt{6}-\frac{\delta }{s}\right)}^{2}}-\sqrt{3}\right)-\frac{2e}{s}\left(\sqrt{2}-\frac{6\left(\sqrt{6}-\frac{\delta }{s}\right)}{\sqrt{9+3{\left(\sqrt{6}-\frac{\delta }{s}\right)}^{2}}}\right)\right)\left(\frac{c}{s}+\sqrt{6}\right)}{\frac{\delta }{s}\left(3\sqrt{3}+\frac{e}{s}2\sqrt{2}\right)}$$

In the proposed kirigami-based hybrid metamaterial, the displacement or force applied at its two ends can be referred to as ‘distant actuation’. The word ‘distant’ describes the ability of the metamaterial to display a change in its shape (depending on the nature of in-plane and out-of-plane Poisson’s ratios) far away from the point of actuation of either displacement or force. Thus, this external far-field actuation enables local shape modulation of the metamaterial based on its microstructural attributes, which can be varied as per the requirement of an application. For the 3D tessellation, similar programmable force-displacement constitutive behavior can also be obtained as in the case of 2D tessellations.

### Summary and perspective

In general, constitutive relations and Poisson’s ratios are fundamental properties of naturally occurring materials, based on which their engineering applications can be designed. The lack of tailorability and restricted margin for such critical properties severely limit the bounds of achievable multi-functional engineering designs using such materials. Over the last decade, the materials community is paying increasing attention to the possibilities of developing artificial material microstructures based on application-specific multi-functional demands, rather than solely relying on the properties of natural materials. There exists a strong rationale to be able to program the constitutive relationships for extreme structural designs, where the capabilities of intrinsic material can be most optimally exploited based on application-specific requirements. In this article, we have shown a range of such unprecedented mechanical properties through kirigami-engineered metamaterials.

Because the fundamental deformation mechanics of the proposed kirigami-based metamaterial is scale-independent, it can be directly utilized for application across micro to macro scales. In this context note that the macroscale material properties in metamaterials are derived from the ‘structure’ at a much lower length-scale (referred to as microscale here), leading to the aspect of developing new ‘materials’ at the higher length-scale with unprecedented physical properties. In such conceptualization of metamaterials, an adequate ‘relative’ difference between the lower and higher length-scales allows us to define the effective physical properties of a ‘material’ (i.e., metamaterial) based on the ‘structure’ therein. Further, the force-displacement constitutive behavior, as presented in this article, can, in principle, be converted to the stress-strain behavior by considering the area of applied force and calculating the corresponding strain. It may be noted here that the proposed unit cell (and the 2D and 3D lattices) under the deformation mode explored in this article are 1° of freedom structures if only kinematic motion is considered before any contact (refer to degree of freedom of a unit cell under consideration in method details). This is advantageous for the microstructural design because it provides more certainty in the global response, given that the proposed kirigami unit cell inherently possesses a rich microstructural design space. Based on the understanding of the pre- and post-contact regimes, as presented in the preceding subsections, we summarize the important contributions of this article in the following paragraphs.

As shown in Figure 3, Figure 4 and 4, a transition from kinematic motion to structural deformation by far-field actuation facilitates programmable stiffness in the proposed metamaterial. Here this transition occurs when edges of adjacent extrusions come in contact, leading to a steep rise in stiffness because of structural deformation. Figure 4 has demonstrated multiple configurations of meta-sheets designed as functions of microstructural geometry for application-centric stiffness modulation. The point of transition for unit-cells in a column or row can be gradually varied by designing meta-sheets with a graded microstructure. For lattice-based cellular metamaterials (Mukhopadhyay and Adhikari, 2017a; Mukhopadhyay et al., 2019b), a lack of mixed-mode deformation with a transition point directly correlates with the unlikeliness of a programmable stiffness having sudden jumps in the value. Scientific studies (Mukhopadhyay et al., 2020a) have been performed to obtain programmable stiffness through contact and structural deformation. However, these studies have focused on compression-based distant actuation, while here, we have also analyzed stiffness modulation while the material is under tension (Figures 3E and 3F). Thus, similar to the compressive loading cases, unique metastructures can be built for tensile loading scenarios by utilizing unit-cells of varying microstructure. Not only that, we demonstrate in the current study that the force-displacement constitutive curve has far more generality in terms of piece-wise modulation of stiffness. It can be noticed in this context that unlike conventional materials, the proposed metamaterial exhibits a programmable non-invariant force-displacement constitutive behavior under compressive and tensile modes. The hinge connections through out-of-plane extrusions of meta-sheets can be utilized to build 3D metastructures comprising both symmetric and asymmetric unit cells. This opens up possibilities of having multidirectional gradation in the microstructure that would be useful in potential application for systems susceptible to multi-modal vibration and multidirectional impacts.

The vastly possible configurations that can be formed using unit-cells with different geometrical attributes expand the capability of these classes of metamaterials dramatically. In Figures 2C–2H and Figures 5G–5I, we have demonstrated deformation-dependent mix-mode (negative and positive values) Poisson’s ratio behavior with its dependence on the microstructure geometry. The transition from positive to negative Poisson’s ratio is shown to vary as a function of the geometric attributes. Thus, by forming 3D metastructures with different unit-cells, the Poisson’s ratio can be extensively modulated in both in-plane and out-of-plane directions. Note that Poisson’s ratios are not only crucial for far-field shape modulation, but they also play a vital role in other mechanical behaviors at the structural level concerning static and dynamic performance. Thus, a capability of Poisson’s ratio programming, with the deformation-dependent constitutive behavior modeling (, and the compound effects thereof) will, in turn, lead to the modulation of such structure-level behavior as well. In summary, the microstructural configuration and extent of applied far-field actuation are key parameters that enable forming a class of metamaterials with programmable shape and stiffness with tunable deformation-dependent multidirectional Poisson’s ratio modulation.

## Conclusions

Through analytical and numerical analyses, supported by elementary-level physical experiments, here we have presented a novel kirigami-inspired hybrid metamaterial with programmable deformation-dependent stiffness and mixed-mode multidirectional auxeticity. In the proposed metamaterial, first symmetric and asymmetric unit cells (i.e., modular units) are developed, the behavior of which is dominated by principles of origami. In the next stage, these modular units are tessellated in kirigami-inspired 2D and 3D domains by designing compatible edge connections. The stiffness and auxeticity of the proposed metamaterials can be modulated as a function of the applied far-field deformation with the microstructural geometric configurations and attributes. A transition from kinematic motion to structural deformation can be programmed to achieve an extreme rise in stiffness in both compressive loading and tensile-loading scenarios as to when in the deformation process it is needed. Through spatially varying graded microstructure with asymmetric geometries, we have further shown that it is possible to modulate piece-wise constitutive curves as per wide-ranging functional demands. This will contribute to advancements in the field of lightweight multi-functional structural systems where the stiffness can be programmed for the most optimum exploitation of the material. It may be noted in this context, unlike conventional materials, the proposed metamaterial exhibits a programmable non-invariant force-displacement constitutive behavior under compressive and tensile modes. The degree of non-invariance can be designed based on microstructural geometry on the basis of application-specific functional demands.

Based on the microstructural attributes, the proposed metamaterial can achieve either pure (only positive or negative) or mixed-mode (both positive and negative value) in-plane and out-of-plane Poisson’s ratios. In addition, the transition between positive and negative out-of-plane Poisson’s ratios during the deformation process can be programmed as a function of the microstructural attributes of the metamaterial. Based on the lateral strain components under applied a far-field displacement (that depends on the programmable multidirectional Poisson’s ratios), large-scale modulations in shape can be obtained in the developed metamaterial, leading to a distant actuation feature without requiring intricate networks of complex locally implanted sensors, actuators, and controllers. In general, the capability of Poisson’s ratio programming in the 2D and 3D spaces, with the deformation-dependent constitutive behavior modeling (, and the compound effects thereof), will lead to the modulation of a range of global structure-level mechanical behavior such as shape morphing, direction-dependent static deformation, vibration, wave propagation, and impact resistance. Such exciting features make the proposed kirigami-based hybrid metamaterial a multi-functionally exploitable crucial addition to the class of innovative programmable metamaterials that can find applications in a wide range of mechanical and aerospace systems, transformable architecture, soft robotics, biomedical equipment and energy absorption structures under multi-directional impact loading.

## STAR★Methods

### Key resources table

**Table:** 

REAGENT or RESOURCE	SOURCE	IDENTIFIER
**Software and algorithms**		

MATLAB R2020a	MathWorks Inc.	https://in.mathworks.com/products/matlab.html; RRID: SCR_001622
ANSYS 18.1	Ansys Inc.	https://www.ansys.com/
MERLIN2	Liu and Paulino, 2017	http://paulino.princeton.edu/software.html

### Resource availability

#### Lead contact

Further information and requests for resources should be directed to and will be fulfilled by the lead contact, Prof. Tanmoy Mukhopadhyay (tanmoy@iitk.ac.in).

#### Materials availability

The study did not generate new unique reagents.

### Method details

#### Geometric configuration and kinematic analysis

##### Geometric configuration

The unit-cell of the proposed kirigami meta-structure is obtained by taking a uniform truncated octahedron of side length s (shown in Figure S1A) as a template. The hexagonal bases in yellow are replaced by prismatic extrusions of length e while prismatic connectors of length c/2 are added to the hexagonal bases shown in green to form the unit-cell (in Figure S1B). The distance between longitudinally opposite sides of a hexagonal base is marked as d. The square components of the truncated octahedron in green remain as is. Thus, the faces in green are rigid while those in yellow are not present in the proposed unit-cell. This yellow and green faceted model of the truncated octahedron helps represent the rigid origami motion of the unit-cell during longitudinal deformation. The lengths and surface angles of the rigid green faces remain unaffected as they only rotate about creases, but the yellow faces get distorted since they are replaced by extrusions. Thus, only the side length of the yellow bases for the extrusions remains preserved, while the surface angles vary as a function of the longitudinal deformation δ.

Figure S1C depicts the side view (ZY Plane) of the undeformed model with relevant dimensions marked. An extrusion that would be part of the unit-cell is represented with grey-coloured line segments in the top left region of the figure for ease in labeling angles. The distance between bases of the connectors is defined as $L_{o}$. The angles for an undeformed truncated octahedron can be found as

(Equation 9) $$\alpha_{o}=120^{o}$$

(Equation 10) $$\beta_{o}=270^{o}-\cos^{-1}\left(\frac{-1}{\sqrt{3}}\right)=144.7356^{o}$$

(Equation 11) $$\theta_{o}=180^{o}-2\;\cos^{-1}\left(\frac{1}{\sqrt{3}}\right)=70.5288^{o}$$

From the basic geometry of a truncated octahedron the distance between the bases of the connectors $\left(L_{o}\right)$ can be found. From Figure S1A, the length d can also be found.

(Equation 12) $$L_{o}=s\sqrt{6}$$

(Equation 13) $$d=2s\;\sin\frac{\alpha }{2}$$

Figure S1D depicts a wire frame model of the unit-cell under a compressive longitudinal deformation of magnitude. Through application of the cosine law we obtain the following relations.

(Equation 14) $${\left(s\sqrt{6}-\delta \right)}^{2}=d^{2}+s^{2}+2ds\;\sin\;\beta$$

(Equation 15) $$s^{2}=d^{2}+{\left(s\sqrt{6}-\delta \right)}^{2}-2d\left(s\sqrt{6}-\delta \right)\sin\;\theta$$

These can be further simplified using Equation (13) to obtain

(Equation 16) $${\left(s\sqrt{6}-\delta \right)}^{2}=s^{2}\left(1+4\;\sin^{2}\frac{\alpha }{2}+4\;\sin\frac{\alpha }{2}\sin\;\beta \right)$$

(Equation 17) $$s^{2}=4s^{2}\;\sin\frac{\alpha }{2}\left(\sin\frac{\alpha }{2}-\left(s\sqrt{6}-\delta \right)\sin\;\theta \right)+{\left(s\sqrt{6}-\delta \right)}^{2}$$

##### Kinematic analysis

Denavit and Hartenberg proposed a matrix method based on the use of four independent parameters, referred to as D-H notations (Denavit (1955)). It provides a straightforward way to reveal the motion of each joint and its relationship with any other joints; so it is adopted to conduct the kinematic analysis (motion behavior) of spatial linkages in this investigation. The setup of each coordinate system in a linkage is presented in Figure S2, where the axis $z_{i}$ is along the revolute joint i, $x_{i}$ is the common normal from $z_{i-1}$ to $z_{i}$, and $y_{i}$ is determined by the right-hand rule. Thus the geometric parameters are defined as the link length $a_{i\left(i+1\right)}$, the link twist $\alpha_{i\left(i+1\right)}$ and the joint offset $R_{i}$, where $a_{i\left(i+1\right)}$ is the normal distance between axes $z_{i}$ and $z_{i+1}$, $\alpha_{i\left(i+1\right)}$ is the angle of rotation from $z_{i}$ to $z_{i+1}$, positive along $x_{i+1}$, and $R_{i}$ is the normal distance between axes $x_{i}$ and $x_{i+1}$, positive along $z_{i}$. The kinematic variable $\theta_{i}$ is defined as the angle of rotation from $x_{i}$ to $x_{i+1}$, positive along $z_{i}$, which measures the rotation between two links joined by the revolute joint $z_{i}$.

For a single-loop linkage consisting of k links, the closure equation is

(Equation 18) $$T_{21}T_{32}\ldots .T_{1K}=I_{4}$$

where the transformation matrix $T_{\left(i+1\right)i}$ is

(Equation 19) $$T_{\left(i+1\right)i}=\left[\begin{array}{cccc} \cos\;\theta_{i} & -\cos\;\alpha_{i\left(i+1\right)}\;\sin\;\theta_{i} & \sin\;\alpha_{i\left(i+1\right)}\;\sin\;\theta_{i} & a_{i\left(i+1\right)}\;\cos\;\theta_{i}\\ \sin\;\theta_{i} & \cos\;\alpha_{i\left(i+1\right)}\;\cos\;\theta_{i} & -\sin\;\alpha_{i\left(i+1\right)}\;\cos\;\theta_{i} & a_{i\left(i+1\right)}\;\sin\;\theta_{i}\\ 0 & \sin\;\alpha_{i\left(i+1\right)} & \cos\;\alpha_{i\left(i+1\right)} & R_{i}\\ 0 & 0 & 0 & 1 \end{array}\right]$$

when i+1>k, it is replaced by 1. It transforms the expression in the coordinate system to the $i_{th}$ coordinate system. The inverse transformation $T_{i\left(i+1\right)}$ has the following property

(Equation 20) $$T_{i\left(i+1\right)}=T_{\left(i+1\right)i}^{-1}=\left[\begin{array}{cccc} \cos\;\theta_{i} & \sin\;\theta_{i} & 0 & -a_{i\left(i+1\right)}\\ -\cos\;\alpha_{i\left(i+1\right)}\;\sin\;\theta_{i} & \cos\;\alpha_{i\left(i+1\right)}\;\cos\;\theta_{i} & \sin\;\alpha_{i\left(i+1\right)} & -R_{i}\;\sin\;\alpha_{i\left(i+1\right)}\\ \sin\;\alpha_{i\left(i+1\right)}\;\sin\;\theta_{i} & -\sin\;\alpha_{i\left(i+1\right)}\;\cos\;\theta_{i} & \cos\;\alpha_{i\left(i+1\right)} & -R_{i}\;\cos\;\alpha_{i\left(i+1\right)}\\ 0 & 0 & 0 & 1 \end{array}\right]$$

As for spherical linkages, the axes intersect at one point as shown in Figure S3, which means the lengths and offsets of each links are zero and thus Equation (18) reduces to

(Equation 21) $$Q_{21}Q_{32}\ldots .Q_{1K}=I$$

where

(Equation 22) $$Q_{\left(i+1\right)i}=\left[\begin{array}{ccc} \cos\;\theta_{i} & -\cos\;\alpha_{i\left(i+1\right)}\;\sin\;\theta_{i} & \sin\;\alpha_{i\left(i+1\right)}\;\sin\;\theta_{i}\\ \sin\;\theta_{i} & \cos\;\alpha_{i\left(i+1\right)}\;\cos\;\theta_{i} & -\sin\;\alpha_{i\left(i+1\right)}\;\cos\;\theta_{i}\\ 0 & \sin\;\alpha_{i\left(i+1\right)} & \cos\;\alpha_{i\left(i+1\right)} \end{array}\right]$$

Subsequently, the inverse transformation is given by

(Equation 23) $$Q_{i\left(i+1\right))}=\left[\begin{array}{ccc} \cos\;\theta_{i} & \sin\;\theta_{i} & 0\\ -\cos\;\alpha_{i\left(i+1\right)}\;\sin\;\theta_{i} & \cos\;\alpha_{i\left(i+1\right)}\;\cos\;\theta_{i} & \sin\;\alpha_{i\left(i+1\right)}\\ \sin\;\alpha_{i\left(i+1\right)}\;\sin\;\theta_{i} & -\sin\;\alpha_{i\left(i+1\right)}\;\cos\;\theta_{i} & \cos\;\alpha_{i\left(i+1\right)} \end{array}\right]$$

In the current investigation, an analysis is performed on the unit linkage loop shown in Figure S4B, which is a part of the larger unit-cell illustrated in Figure S4A for reference. The unit linkage loop has 3 mountain creases and 2 valley creases. Axes $x_{2}$, $x_{3}$ and kinematic variable $\theta_{2}$ are shown in Figure S4B, while the remaining parameters are determined in a similar fashion. Since the linkage loop under consideration has 5 links, Equation (21) can be written as,

(Equation 24) $$Q_{54}Q_{15}Q_{21}=Q_{34}Q_{23}$$

where,

(Equation 25) $$Q_{54}=\left[\begin{array}{ccc} \cos\;\theta_{4} & 0 & \sin\;\theta_{4}\\ \sin\;\theta_{4} & 0 & -\cos\;\theta_{4}\\ 0 & 1 & 0 \end{array}\right]$$

(Equation 26) $$Q_{15}=\left[\begin{array}{ccc} \cos\;\theta_{5} & 0 & \sin\;\theta_{5}\\ \sin\;\theta_{5} & 0 & -\cos\;\theta_{5}\\ 0 & 1 & 0 \end{array}\right]$$

(Equation 27) $$Q_{21}=\left[\begin{array}{ccc} \cos\;\theta_{1} & 0 & \sin\;\theta_{1}\\ \sin\;\theta_{1} & 0 & -\cos\;\theta_{1}\\ 0 & 1 & 0 \end{array}\right]$$

(Equation 28) $$Q_{34}=\left[\begin{array}{ccc} \cos\;\theta_{3} & \sin\;\theta_{3} & 0\\ 0 & 0 & 1\\ \sin\;\theta_{3} & -\cos\;\theta_{3} & 0 \end{array}\right]$$

(Equation 29) $$Q_{23}=\left[\begin{array}{ccc} \cos\;\theta_{2} & \sin\;\theta_{2} & 0\\ 0 & 0 & 1\\ \sin\;\theta_{2} & -\cos\;\theta_{2} & 0 \end{array}\right]$$

Substituting these matrices in Equation (24) we get the following nine relations

(Equation 30) $$\cos\;\theta_{3}\;\cos\;\theta_{2}=\cos\;\theta_{4}\;\cos\;\theta_{5}cos\theta_{1}+\sin\;\theta_{4}\;\sin\;\theta_{1}$$

(Equation 31) $$\cos\;\theta_{3}\;\sin\;\theta_{2}=\cos\;\theta_{4}\;\sin\;\theta_{5}$$

(Equation 32) $$\sin\;\theta_{3}=\cos\;\theta_{4}\;\cos\;\theta_{5}\;\sin\;\theta_{1}-\sin\;\theta_{4}\;\cos\;\theta_{1}$$

(Equation 33) $$\sin\;\theta_{2}=\cos\;\theta_{5}\;\cos\;\theta_{1}\;\sin\;\theta_{4}-\cos\;\theta_{4}\;\sin\;\theta_{1}$$

(Equation 34) $$-\cos\;\theta_{2}=\sin\;\theta_{4}\;\sin\;\theta_{5}$$

(Equation 35) $$\cos\;\theta_{5}\;\sin\;\theta_{1}\;\sin\;\theta_{4}=-\cos\;\theta_{4}\;\cos\;\theta_{1}$$

(Equation 36) $$\cos\;\theta_{2}\;\sin\;\theta_{3}=\cos\;\theta_{1}\;\sin\;\theta_{5}$$

(Equation 37) $$\sin\;\theta_{3}\;\sin\;\theta_{2}=-\cos\;\theta_{5}$$

(Equation 38) $$-\cos\;\theta_{3}=-\sin\;\theta_{5}\;\sin\;\theta_{1}$$

These nine equations can be simplified to the following 3 relations

(Equation 39) $$\tan\;\theta_{2}=-\cot\;\theta_{5}\;\sec\;\theta_{1}$$

(Equation 40) $$\cos\;\theta_{3}=-\sin\;\theta_{5}\;\sin\;\theta_{1}$$

(Equation 41) $$\tan\;\theta_{4}=-\cot\;\theta_{1}\;\sec\;\theta_{5}$$

Generally, in origami study, dihedral angles are preferred to directly represent the folding process. Dihedral angles are the angles between adjacent faces of an origami fold and are depicted in Figure S4D. Relations can be found between Kinematic variables and Dihedral angles

(Equation 42) $$\varphi_{1}=180^{o}-\theta_{1}$$

(Equation 43) $$\varphi_{2}=180^{o}-\theta_{2}$$

(Equation 44) $$\varphi_{3}=\theta_{3}-180^{o}$$

(Equation 45) $$\varphi_{4}=180^{o}-\theta_{4}$$

(Equation 46) $$\varphi_{5}=180^{o}-\theta_{5}$$

We can now reframe Equations (39), (40) and (41) in terms of dihedral angles as

(Equation 47) $$\tan\;\varphi_{2}=\cot\;\varphi_{5}\;\sec\;\varphi_{1}$$

(Equation 48) $$\cos\;\varphi_{3}=\sin\;\varphi_{5}\;\sin\;\varphi_{1}$$

(Equation 49) $$\tan\;\varphi_{4}=\cot\;\varphi_{1}\;\sec\;\varphi_{5}$$

These dihedral angles can be expressed in terms of the angles α, β and θ denoted in Figure S1B. The required expressions are mentioned below:

(Equation 50) $$\varphi_{2}=\alpha$$

(Equation 51) $$\varphi_{4}=360^{o}-\beta$$

(Equation 52) $$\varphi_{5}=180^{o}-\frac{\alpha }{2}$$

From these, we get the following relation between $\varphi_{2}$ and $\varphi_{5}$

(Equation 53) $$\varphi_{2}+2\varphi_{5}=360^{o}$$

We now reframe Equations (47), (48), and (49) to express them in terms of only $\varphi_{5}$

(Equation 54) $$\sec\;\varphi_{1}=-\tan\;2\varphi_{5}\;\tan\;\varphi_{5}$$

(Equation 55) $$\cos\;\varphi_{3}=\pm \sin\;\varphi_{5}\sqrt{1-\cot^{2}\varphi_{5}\;\cot^{2}2\varphi_{5}}$$

(Equation 56) $$\tan\;\varphi_{4}=\frac{\pm \sec\;\varphi_{5}}{\sqrt{\tan^{2}\varphi_{5}\;\tan^{2}2\varphi_{5}-1}}$$

We can replace $\varphi_{4}$ with $360^{o}\beta$ and $\varphi_{5}$ with $180^{o}\frac{\alpha }{2}$ in Equation (56):

(Equation 57) $$\tan\;\beta =\frac{\pm \sec\frac{\alpha }{2}}{\sqrt{\tan^{2}\;\alpha \tan^{2}\frac{\alpha }{2}-1}}$$

This can be further simplified to obtain a single relation

(Equation 58) $$\sin\;\beta =\frac{-\cos\;\alpha }{\sin\frac{\alpha }{2}}$$

Thus a relation between α and β has been found. We now further simplify the Equation (16) and (17) that have been formulated using Geometrical Analysis. Eliminating β from Equation (16) using Equation (58) we obtain

(Equation 59) $$\alpha =2\;\sin^{-1}\left(\sqrt{\frac{{\left(s\sqrt{6}-\delta \right)}^{2}+3s^{2}}{12s^{2}}}\right)$$

Equation (59) can be used to eliminate α from Equation (17)

(Equation 60) $$\theta =\sin^{-1}\left(\frac{2\left(s\sqrt{6}-\delta \right)}{\sqrt{9s^{2}+3{\left(s\sqrt{6}-\delta \right)}^{2}}}\right)$$

Similarly β can be expressed as

(Equation 61) $$\beta =\sin^{-1}\left(\frac{{\left(s\sqrt{6}-\delta \right)}^{2}-3s^{2}}{s\sqrt{9s^{2}+3{\left(s\sqrt{6}-\delta \right)}^{2}}}\right)$$

Thus we have obtained expressions for each of the angles α, β and θ in terms of a given axial displacement δ and the side length s.

#### Description of structural analysis for the kirigami based metamaterial

In this work, deformation of the kirigami based metamaterial is simulated using a bar and hinge based idealized structural model as shown in Figure S5 (Liu and Paulino (2016)). The triangular facet is modeled by bar elements, whereas the crease is modeled by an equivalent rotational spring stiffness in this idealization. The equilibrium equation for an idealized bar-hinge based model can be expressed as (Liu and Paulino (2016)).

(Equation 62) $$\frac{\partial U_{bar}}{\partial u}+\frac{\partial U_{spring}}{\partial u}=F$$

where $U_{bar}$ and $U_{spring}$ denote the strain energy stored in the bars and the spring, respectively, while u represents the nodal displacement vector. It can be noted that the stored energy in the system would depend on the axial stiffness of the bars EA, the rotational stiffness of the springs along fold lines $K_{f}$ and bending stiffness of facets $K_{b}$ (Liu and Paulino (2016)). The above equation is solved following the modified generalized displacement control method Liu and Paulino (2016), which is an arc-length type method leading to the whole equilibrium path of the displacement-controlled system.

We have carried out the analysis in multiple stages. Initially we allow the folding-induced deformation along the creases. When contact of the prismatic extrusions occurs during longitudinal deformation of the unit-cell, the movement of the contacted nodes are restrained to capture the structural deformation and the entire simulation is performed again, considering the contacted shape as the initial configuration.

As per the current methodology, three stiffness components are involved in a generic origami structure: folding stiffness along creases $\left(K_{f}\right)$, bending stiffness along the line of bending in facets $\left(K_{b}\right)$ and axial stiffness of the bars (EA).To obtain the structural analysis plots presented in Figure 2, Figure 3, Figure 4 of the main text, the geometrical parameters for the physical specimen are adopted in the numerical model. The bar axial stiffness, bar bending stiffness, and spring rotational stiffness per unit length are considered as $EA=1\times 10^{4}N$, $K_{b}=1\times 10^{3}N$ and $K_{f}=1\times 10^{-1}N$.

Note here that $K_{b}$ is the stiffness per unit length of the rotational spring in each panel to enable bending while $K_{f}$ is the stiffness per unit length of the rotational springs along crease lines to enable folding. In general, the stiffness has a unit of N-m. Since $K_{b}$ and $K_{f}$ are defined here as stiffness per unit length, these have the unit N. The axial stretching stiffness EA is the linear spring stiffness along a bar with a unit of N. These bars define a facet and can be seen in Figure S5.

The analysis presented in this section is general and it takes a non-rigid origami approach by assigning bending stiffness $\left(K_{b}\right)$ and folding stiffness $\left(K_{f}\right)$ to origami panels. For a rigid origami assumption, we have assigned a very low magnitude to $K_{f}$ while assigning a very large magnitude to $K_{f}$ (such that $K_{b}/K_{f}∼$ 10^4^). This practically eliminates bending in panels and allows the creases to fold relatively much easily. This can be confirmed from the plots in Figures 2A and 2B of the main text, where the results from geometric analysis and the idealized structural simulation with the above-mentioned conditions agree perfectly.

Note that based on the structural analysis methodology described above, the equivalent force-displacement constitutive behavior of each unit-cell (pre- and post-contact) can be obtained. Subsequently, a spring (in series and parallel) based analogy can be adopted to obtain the force-displacement curves of the proposed metamaterial by idealizing it as a network of equivalent springs. In such analyses, we consider a displacement-controlled simulation with the assumption of two parallel edges during any stage of deformation, while the vertical displacement is applied perpendicular to these horizontal (and parallel) edges.

#### Poisson’s ratio of the kirigami based metamaterial

Poisson’s ratio can usually be reported in two ways: I. Tangent Poisson’s ratio (calculated by taking derivative at each instance of the deformation path) and II. Secant Poisson’s ratio (calculated based on the cumulative deformations between two distinct points in the deformation path). One may note that the definition for tangent Poisson’s ratio and secant Poisson’s ratio stated here are similar to Poisson’s function and Poisson’s ratio defined in Smith et al. (1999), respectively. The main emphasis of this paper is on secant Poisson’s ratio. However, relevant analytical expressions to obtain the tangent Poisson’s ratio for the proposed metamaterial are provided here for extensiveness.

##### In-plane Poisson’s ratio $\left(\nu_{zy}\right)$

Figure S6A depicts the top view (XY plane) of the metamaterial with horizontally adjacent cells attached through hinged connections along the edges of their in-plane prismatic extrusions (marked in green). The two out-of-plane extrusions shown in the unit-cell in Figure S6B are replaced with hexagonal faces (in yellow) at their bases in Figure S6C for better visualization during analysis. The in-plane horizontal and vertical dimensions of the unit-cell are shown as H and V, respectively. The horizontal dimension H can be expressed as a sum of $h_{1}$, $h_{2}$ and $h_{3}$ as shown.

(Equation 63) $$h_{1}=e\;\sin\left(\theta \right)$$

(Equation 64) $$h_{2}=s\;\cos\left(270-\theta -\beta \right)$$

(Equation 65) $$h_{3}=s\frac{\sqrt{3}}{2}$$

Thus, we can express the horizontal length (H) as:

(Equation 66) $$H=s\left[\sqrt{3}-2\left(\sin\;\theta \;\cos\;\beta +\cos\;\theta \;\sin\;\beta \right)\right]+2e\;\sin\;\theta$$

Through Equations (60) and (61) we can obtain an expression for H as a function of the longitudinal deformation δ and the geometric attributes s, c and e:

(Equation 67) $$H=s\left(\sqrt{3}+\frac{2\sqrt{9s^{2}-{\left(s\sqrt{6}-\delta \right)}^{2}}}{3s}\right)+\frac{4e\left(s\sqrt{6}-\delta \right)}{\sqrt{9s^{2}+3{\left(s\sqrt{6}-\delta \right)}^{2}}}$$

Similarly, the vertical length (V) and the vertical length at no longitudinal deformation $\left(V_{o}\right)$ can be deduced from Figure S6C as:

(Equation 68) $$V=2c+s\sqrt{6}-\delta$$

(Equation 69) $$V_{o}=2c+s\sqrt{6}$$

The horizontal length for no longitudinal deformation $\left(H_{o}\right)$ and the change in horizontal length can be found using Equation (67) as:

(Equation 70) $$H_{o}=s\frac{5\sqrt{3}}{3}+e\frac{4\sqrt{2}}{3}$$

(Equation 71) $$\Delta H=s\left(\frac{2}{\sqrt{3}}-\frac{2\sqrt{9-{\left(\sqrt{6}-\frac{\delta }{s}\right)}^{2}}}{3}\right)+4e\left(\frac{\sqrt{2}}{3}-\frac{\left(\sqrt{6}-\frac{\delta }{s}\right)}{\sqrt{9+3{\left(\sqrt{6}-\frac{\delta }{s}\right)}^{2}}}\right)$$

The secant Poisson’s ratio is defined as:

(Equation 72) $$\nu =-\frac{\Delta H/H_{o}}{\Delta V/V_{o}}$$

Thus the in-plane secant Poisson’s ratio for the proposed unit-cell can be obtained from the above equations as:

(Equation 73) $$\nu_{zy}=\frac{\left(\left(\frac{2}{3}\sqrt{9-{\left(\sqrt{6}-\frac{\delta }{s}\right)}^{2}}-\frac{2}{\sqrt{3}}\right)-\frac{4e}{s}\left(\frac{\sqrt{2}}{3}-\frac{\left(\sqrt{6}-\frac{\delta }{s}\right)}{\sqrt{9+3{\left(\sqrt{6}-\frac{\delta }{s}\right)}^{2}}}\right)\right)\left(\frac{6c}{s}+3\sqrt{6}\right)}{\frac{\delta }{s}\left(5\sqrt{3}+\frac{e}{s}4\sqrt{2}\right)}$$

The tangent Poisson’s ratio is defined as:

(Equation 74) $$\nu^{t}=-\frac{dH/H}{dV/V}$$

One can obtain the tangent Poisson’s ratio using Equations (67), (68), and (74). Closed-form expression of the tangent Poisson’s ratio can also be readily obtained using these expressions.

##### Out-of-plane Poisson’s ratio $\left(\nu_{zx}\right)$

Figure S7A depicts the top view (XY plane) of the metamaterial with unit-cells attached through hinged connections along the edges of their out-of-plane prismatic extrusions (marked in green). Figure S7B depicts the side view (XZ plane) with the hinged connections marked. The out-of-plane horizontal extent of the unit-cell is shown as W in Figure S7C, while the vertical extent remains the same as that defined in section SM3.1. The horizontal extent W can be expressed as a sum of s, $w_{1}$ and $w_{2}$ as shown. From the geometry of a hexagon we can find the following relations:

(Equation 75) $$w_{1}=h_{1}\frac{\sqrt{3}}{2}=e\;\sin\left(\theta \right)\frac{\sqrt{3}}{2}$$

(Equation 76) $$w_{2}=h_{2}\frac{\sqrt{3}}{2}=s\;\cos\left(270-\theta -\beta \right)\frac{\sqrt{3}}{2}$$

Through Equations (60) and (61) we can obtain an expression for W as a function of the longitudinal deformation δ and the geometric attributes s, c and e:

(Equation 77) $$W=s\left(2+\frac{\sqrt{9s^{2}-{\left(s\sqrt{6}-\delta \right)}^{2}}}{\sqrt{3}s}\right)+\frac{2\sqrt{\left(3\right)}e\left(s\sqrt{6}-\delta \right)}{\sqrt{9s^{2}+3{\left(s\sqrt{6}-\delta \right)}^{2}}}$$

The horizontal length for no longitudinal deformation $\left(W_{o}\right)$ and the change in horizontal length can be found using Equation (77) as:

(Equation 78) $$W_{o}=3s+\sqrt{\frac{8}{3}}e$$

(Equation 79) $$\Delta W=s\left(1-\sqrt{3-\frac{{\left(\sqrt{6}-\frac{\delta }{s}\right)}^{2}}{3}}\right)+\frac{2e}{\sqrt{3}}\left(\sqrt{2}-\frac{6\left(\sqrt{6}-\frac{\delta }{s}\right)}{\sqrt{9+3{\left(\sqrt{6}-\frac{\delta }{s}\right)}^{2}}}\right)$$

The secant Poisson’s ratio is defined as:

(Equation 80) $$\nu =-\frac{\Delta W/W_{o}}{\Delta V/V_{o}}$$

Thus the out-of-plane secant Poisson’s ratio for the proposed unit-cell can be obtained from the above equations as:

(Equation 81) $$\nu_{zx}=\frac{\left(\left(\sqrt{9-{\left(\sqrt{6}-\frac{\delta }{s}\right)}^{2}}-\sqrt{3}\right)-\frac{2e}{s}\left(\sqrt{2}-\frac{6\left(\sqrt{6}-\frac{\delta }{s}\right)}{\sqrt{9+3{\left(\sqrt{6}-\frac{\delta }{s}\right)}^{2}}}\right)\right)\left(\frac{c}{s}+\sqrt{6}\right)}{\frac{\delta }{s}\left(3\sqrt{3}+\frac{e}{s}2\sqrt{2}\right)}$$

The tangent Poisson’s ratio is defined as:

(Equation 82) $$\nu^{t}=-\frac{dW/W}{dV/V}$$

One can obtain the tangent Poisson’s ratio for out-of-plane direction using Equations (77), (68), and (82). Closed-form expression of the tangent Poisson’s ratio can also be readily obtained using these expressions.

#### Contact induced stiffness modulation

##### Microstructural geometry upon contact and initial constraints

Initially, under far-field actuation, the metamaterial undergoes a purely rigid deformation (i.e., deformation due to folding along the creases only) until contact among edges occurs. At this point, the facets of the unit-cell must deform to allow any further vertical deformation. Therefore, non-rigid behavior is observed in the post-contact regime. Here we have obtained closed-form expressions to quantify the longitudinal deformation required for initial contact in a unit-cell in terms of its geometric attributes. Figure S8A depicts the state of a portion of the metamaterial just after contact under compressive loading. The angle θ (shown in Figure S1B) is defined in this state of contact as $\theta_{cc}$. From the geometric configuration at contact we get

(Equation 83) $$\theta_{cc}=\cos^{-1}\left(\frac{c}{2e}\right)$$

Equations (60) and (83) are used to obtain an expression for the longitudinal deformation required for contact in compressive loading $\left(\delta_{cc}\right)$:

(Equation 84) $$\delta_{cc}=s\left(\sqrt{6}-3\sqrt{\frac{1-{\left(\frac{c}{2e}\right)}^{2}}{1+3{\left(\frac{c}{2e}\right)}^{2}}}\right)$$

Figure S8B depicts the state of a portion of the metamaterial just after contact under tensile loading. The angle θ (shown in Figure S8B) has a value of $90^{o}$ in this state. Equation (60) is used to obtain an expression for the longitudinal deformation required for contact in tensile loading $\left(\delta_{ct}\right)$:

(Equation 85) $$\delta_{ct}=s\left(3-\sqrt{6}\right)$$

Since we aim to achieve a transition from rigid to non-rigid deformation through contact, here we have found expressions that set certain constraints on the geometric attributes c and e. From Figure S8A it can be deduced that the geometric configuration of a unit-cell should be such that the angle $\theta_{o}$ in the undeformed state (Equation 11) must be more than $\theta_{cc}$. It can also be observed that when contact takes place (in compressive loading) a triangular configuration is formed between the extrusions of vertically adjacent unit-cells and their connector. Thus through triangle inequality and the constraint on $\theta_{o}$, the following expression can be found:

(Equation 86) $$\frac{1}{3}<\frac{c}{2e}<1$$

##### Requirement for 'asymmetric-I′ and 'asymmetric-II′ unit-cells

We have introduced two types of asymmetric unit-cells known as the 'Asymmetric-I′ and the 'Asymmetric-II′ unit-cells. The Asymmetric-I unit-cell is obtained by having two pairs of diametrically opposite extrusions of length $e_{2}$ and the remaining pair of length $e_{1}$. Here the in-plane extrusions are of length $e_{1}$ while the out-of-plane extrusions are of length $e_{2}$. The Asymmetric-I unit-cell facilitates gradation in a tessellated structure, which in turn facilitates programmable stiffness. The Asymmetric-II unit-cell has two of the out-of-plane extrusions of identical lengths as the in-plane extrusions $\left(e_{1}\right)$ while the remaining two out-of-plane extrusions of length $e_{2}$. The Asymmetric-II unit-cell enables subtle variations in the graded structures for added programmability.

Consider the 'Configuration 1′ described in Figure 4B of the main text. Column-wise gradation in a 2D metasheet is achieved through Asymmetric-I unit-cells. A 'set' was initially defined as a group of consecutive unit-cells with the same geometry, attached longitudinally through their connectors. The gradation is achieved through changes in these 'sets' along a column, with each successive set consisting of unit-cells with smaller $e_{2}$ magnitudes as compared to the previous set. Configuration 1 was obtained by varying $e_{2}$ from 2.6 to 2.0 in each column. This variation in $e_{2}$ directly relates to a variation in the vertical displacement required for unit-cells in each set to make contact and henceforth increase stiffness.

One may note that for edge-to-edge contact between vertically adjacent unit-cells in compression, the $e_{2}$ values must be the same. If the $e_{2}$ values are different, contact could take place on the facets of extrusions rather than the edges. This does not pose a problem for contact within each set since the unit-cells are of similar geometry. However, at the interface between successive sets, two unit-cells with different $e_{2}$ would be making contact. To prevent this from occurring, the Asymmetric-II unit-cell was introduced. Among the four out-of-plane extrusions in an Asymmetric-I unit-cell, one pair points downward while the other pair points upward. Thus, the Asymmetric-II unit-cell was formed from the Asymmetric-I unit-cell by fixing the length of one pair of extrusions as $e_{1}$ and the other pair as $e_{2}$. This enables proper edge-to-edge contact at an interface.

For a detailed explanation, we have presented the formation of an interface between two sets in Figure S9. For all unit-cells, connector length (c) and side length (s) are fixed as 2.0 and 1.0, respectively. The in-plane parameter $e_{1}$ of all unit-cells is fixed as 2.0. Figures S9A and S9B depict Asymmetric-I unit-cells with out-of-plane extrusion lengths $e_{2A}$ (in brown) and $e_{2B}$ (in green), respectively. Figure S9C depicts an Asymmetric-II unit-cell with a pair of upward pointing out-of-plane extrusions of length $e_{2A}$. Figure S9D depicts an Asymmetric-II unit-cell with a pair of upward pointing out-of-plane extrusions of length $e_{2B}$. Both the unit-cells in Figures S9C–S9D have a pair of downward pointing out-of-plane extrusions of length $e_{1}$ (in yellow). To depict the gradation in Configuration 1 from Figure 4B of the main text, we have assigned values of 2.6 and 2.5 to $e_{2A}$ and $e_{2B}$, respectively.

To enable proper contact between unit-cells at an interface we defined that a set would have Asymmetric-II unit-cells at its extreme ends. A set of 'n' members would therefore consist of two Asymmetric-II unit-cells at the ends and n−2 Asymmetric-I unit-cells in between. Figure S9E depicts the configuration that would form at an interface between sets with out-of-plane extrusion lengths $e_{2A}$ (set-1) and $e_{2B}$ (set-2). The top most unit-cell is the one shown in Figure S9A, representing set-1. The second unit-cell from the top is the Asymmetric-II unit-cell of set-1, which is also shown in Figure S9C. The third unit-cell from the top is the Asymmetric-II unit-cell of set-2, which is also shown in Figure S9D. Note that the orientation of the unit-cell was flipped for the proper contact at the interface. Finally, the bottom-most unit-cell is the one shown in Figure S9B, representing set-2. Now, one can note that out-of-plane extrusions of the same length $\left(e_{1}\right)$ are present at the interface and depicted in yellow. This ensures that edge-to-edge contact would occur at a vertical displacement obtained from Equation (84) for s=1.0, c=2.0, and $e=e_{1}=2.0$. The other pair of out-of-plane extrusions in these unit-cells have the same length as the set they belong to, ensuring proper contact within their sets too. Thus, in this manner, the Asymmetric-II and Asymmetric-I unit-cells enable gradation in 2D tessellations.

##### Structural analysis of 'asymmetric-I′ unit-cells

An 'asymmetric-I′ unit-cell (shown in Figure 1C of main text) is obtained by having two pairs of diametrically opposite extrusions of length $e_{2}$ and the remaining pair of length $e_{1}$ while the connector remains the same. The extrusions of length $e_{1}$ are said to be in-plane while the others are out-of-plane. In the case of compressive loading of symmetric unit-cells, both the in-plane and out-of-plane extrusions make contact at an equal amount of longitudinal deformation. This leads to a single 'jump' in stiffness as shown in Figure 3 of the main text. However, in the case of 'asymmetric-I′ unit-cells, depending on whether the out-of-plane extrusions are longer or shorter than the in-plane extrusions, they will make contact earlier or later, respectively. This dual contact behavior in an 'asymmetric-I′ unit-cell will lead to two 'jumps' in stiffness as compared to the single jump in stiffness of symmetrical unit-cells. Here we describe the structural analysis performed to obtain constitutive relations for individual asymmetric-I unit-cells.

Figure S10 depicts the constitutive relation for an asymmetric-I unit-cell with the geometric attributes s=1, c=2, $e_{1}=2$ and $e_{2}=2.6$. For the unit-cell under consideration, it can be found that first contact (due to out-of-plane extrusions) occurs at δ=0.1448 while the second contact (due to in-plane extrusions) occurs at δ=0.4855. The same can be verified from Figure S10. Thus, we have carried out the analysis in multiple stages. The first section corresponds to rigid origami motion with folding-induced deformation along the creases, which provides almost negligible stiffness. When contact of the prismatic extrusions occurs, the movement of contacted nodes is restrained, to capture the structural deformation and the entire simulation is performed again considering the contacted shape as the initial configuration of the unit-cell. The force output (load) is obtained for unit-cells of different geometric attributes in this manner to form a graded meta sheet as described in Figure 4 of the main text. The idealized unit-cells in a meta-sheet are analogous to a lattice structure made from a mechanical spring network in series and parallel connections. Thus, a similar approach is followed using the spring network analogy in obtaining the equivalent stiffness of the meta-sheet as a function of compressive vertical deformation (δ) at each stage.

#### Qualitative experiments based on finite element simulations and physical prototypes

The lattice level constitutive relations of the proposed kirigami-inspired metamaterial depend on the elementary unit-cell level mechanical behavior. Thus, we focus on a qualitative comparative assessment of the unit cells in this section. We have considered three different methods of analysis to investigate the comparative deformation behavior, ideal structural simulation, finite element simulation using ANSYS, and behavior of a physical prototype (refer to Figure S11).

To investigate the longitudinal stiffness of the symmetric unit-cell, a specimen made from 220 GSM paper sheet of 2.54 mm in thickness was constructed and compressed in the longitudinal direction. The geometric parameters of the unit-cell were selected as follows: s=20 mm, e=40 mm, and c=40 mm. The crease lines were folded by hand, and a standard poly-synthetic resin based adhesive was used for fabrication purposes. Consider the unit-cell in Figure S1B. The lower edges of the bottom connector were restrained against vertical movement, while a compressive longitudinal load was applied on the upper edges of the top connector. A standard digital camera was used for recording deformation purposes (refer to Video S1). The deformation process of the unit-cell at different stages is presented in Figure S11. Figure S11A depicts the undeformed state of the unit-cell, while Figure S11B and S11C depict two different stages as the deformation progress.

We also modeled the deformation process of the unit cell using ANSYS static structural for a qualitative comparison. A CAD model was designed first for the analysis. The geometric parameters of the unit-cell were selected as follows: s=20 mm, e=40 mm, and c=40 mm. A tetrahedral meshing scheme was utilized with SOLID187 type elements. The material was assigned with the following properties: density = 7850 kg/m^3^, elastic modulus = $2\times 10^{11}$ N/m^2^, Poisson’s Ratio = 0.3. The lower edges of the bottom connector were restrained against vertical movement, while a compressive longitudinal load was applied on the upper edges of the top connector. The load was applied in a ramped mode and has a magnitude of 35,000 N. Figures S11E and S11F depict similar deformed shapes as that of the physical model corresponding to two different stages. Additionally, we have also included two similar stages of deformation obtained from the idealized structural simulation (refer to description of structural analysis for the kirigami based metamaterial in method details) in Figures S11G–S11I for a qualitative comparison. Figure S11G depicts the undeformed state of the unit-cell, while Figures S11H and S11I depict two similar intermediate stages as shown for the physical model and the ANSYS based FEA. It can be noted that all the three forms of analysis show excellent qualitative resemblance in the deformation behavior at the elementary unit cell level, as evident from Figure S11 and Video S1. The qualitative analysis, along with the quantitative results presented in Figures 2A and 2B of the main text, provide adequate confidence in the lattice-level numerical results presented in this article.

#### Degree of freedom of a unit cell under consideration

In this section we show that the unit cell under consideration has 1° of freedom (DOF). This is also tantamount to having 1° of freedom in the lattice structures before any contact. As explained in microstructural geometric configuration of the main text, the truncated octahedron unit cell consists of eight hexagonal faces and 6 square faces. Three pairs of the diametrically opposite hexagonal face are 'open' (flexible) and replaced with prismatic extrusions, which enables their hexagonal shape to be deformable. The remaining pair of hexagonal faces are only allowed to displace longitudinally with respect to one another, implying that their shapes remain as hexagons throughout deformation. The six square faces are rigid and must retain their shape throughout deformation. Three square faces share a side, each with the top hexagonal panel, while three share a side, each with the bottom hexagonal panel, leading to zero transitional DOF for the longitudinal loading. One may consider these square faces as 'flaps' to the top and bottom hexagonal panels. However, each square panel can rotate about the shared side, leading to one rotational DOF per square flap. Additionally, the bottom hexagonal panel can translate toward or away from the top one, giving one DOF. Note that the remaining hexagon faces are flexible and do not have independent DOF. So, in total, we have (before rigid constraints)

(Equation 87) 6DOF(Flaps)+1DOF(LongitudinalTranslation)=7DOF

We also must consider ‘side-length’ constraints to keep the overall structure rigid. Each flexible hexagon can change its shape, but its side lengths must remain the same as in the original unit-cell. There are three such side length constraints per hexagon, but one of these sides is shared with either the top or bottom hexagonal panels. Therefore, we have two side length constraints per flexible hexagon, and each such constraint is paired with two other flexible hexagons, leading to

(Equation 88) $$\frac{\left(6\;flexible\;hexagons\right)\times \left(2\;constraints\;per\;hexagon\right)}{2\;hexagons\;per\;constraint}=6\;rigid\;constraints$$

So, in total, we have 1 DOF for the unit-cell. This simple analysis is consistent with the derived result in Equations (1), (2), and (3) of the main text.

## Data Availability

• All data reported in this paper will be shared by the lead contact upon request. • This paper does not report original code. • Any additional information required to reanalyze the data reported in this paper is available from the lead contact upon request. • All datasets used to generate the results are available in the main article. Further details could be obtained from the corresponding author on reasonable request.

## Supplemental information

Video S1. Qualitative comparison of deformation behaviour, related to “validation of elementary unit cell kinematics” of the main text Qualitative experiments at the unit cell level, refer to Figure 1.

## References

[bib1] Alderson K.L., Fitzgerald A., Evans K.E. (2000). The strain dependent indentation resilience of auxetic microporous polyethylene. J. Mater. Sci..

[bib2] Allen T., Shepherd J., Hewage T., Senior T., Foster L., Alderson A. (2015). Low-kinetic energy impact response of auxetic and conventional open-cell polyurethane foams. physica status solidi.

[bib3] Babaee S., Shim J., Weaver J.C., Chen E.R., Patel N., Bertoldi K. (2013). 3d soft metamaterials with negative Poisson’s ratio. Adv. Mater..

[bib4] Babaee S., Shim J., Weaver J.C., Chen E.R., Patel N., Bertoldi K. (2013). Metamaterials: 3d soft metamaterials with negative Poisson’s ratio. Adv. Mater..

[bib5] Barbarino S., Bilgen O., Ajaj R.M., Friswell M.I., Inman D.J. (2011). A review of morphing aircraft. J. Intell. Mater. Syst. Struct..

[bib6] Callens S.J., Tümer N., Zadpoor A.A. (2019). Hyperbolic origami-inspired folding of triply periodic minimal surface structures. Appl. Mater. Today.

[bib7] Charkhabi S., Chan Y.J., Hwang D., Frey S.T., Bartlett M.D., Reuel N.F. (2019). Kirigami-enabled, passive resonant sensors for wireless deformation monitoring. Adv. Mater. Technol..

[bib8] Choi J., Lakes R. (1991). Design of a fastener based on negative Poisson’s ratio foam. Cell. Polym..

[bib9] Choi J.B., Lakes R.S. (1992). Non-linear properties of polymer cellular materials with a negative Poisson’s ratio. J. Mater. Sci..

[bib10] Christensen J., Kadic M., Wegener M., Kraft O., Wegener M. (2015). Vibrant times for mechanical metamaterials. MRS Communications.

[bib11] Danesh F., Baghi A., Kalantari S. (2018).

[bib12] Denavit J. (1955).

[bib13] Dureisseix D. (2012). An overview of mechanisms and patterns with origami. Int. J. Space Struct..

[bib14] Evans K.E., Alderson A. (2000). Auxetic materials: functional materials and structures from lateral thinking. Adv. Mater..

[bib15] Evans K.E., Nkansah M.A., Hutchinson I.J., Rogers S.C. (1991). Molecular network design. Nature.

[bib16] Fu H., Nan K., Bai W., Huang W., Bai K., Lu L., Zhou C., Liu Y., Liu F., Wang J. (2018). Morphable 3d mesostructures and microelectronic devices by multistable buckling mechanics. Nat. Mater..

[bib17] Fu M., Liu F., Hu L. (2018). A novel category of 3d chiral material with negative Poisson’s ratio. Compos. Sci. Technol..

[bib18] Gao Q., Wang L., Zhou Z., Ma Z., Wang C., Wang Y. (2018). Theoretical, numerical and experimental analysis of three-dimensional double-v honeycomb. Mater. Des..

[bib19] Ghuku S., Mukhopadhyay T. (2022). Anti-curvature honeycomb lattices for mode-dependent enhancement of nonlinear elastic properties under large deformation. Int. J. Non Lin. Mech..

[bib20] Gibson L., Ashby M. (1999).

[bib21] Grima J.N., Gatt R. (2010). Perforated sheets exhibiting negative Poisson’s ratios. Adv. Eng. Mater..

[bib22] Ha C.S., Plesha M.E., Lakes R.S. (2016). Chiral three-dimensional lattices with tunable Poisson’s ratio. Smart Mater. Struct..

[bib23] Haque A.B.M.T., Hwang D., Bartlett M.D. (2021).

[bib24] He Y.L., Zhang P.W., You Z., Li Z., Wang Z., Shu X. (2020). Programming mechanical metamaterials using origami tessellations. Compos. Sci. Technol..

[bib25] Hwang D.G., Bartlett M.D. (2018). Tunable mechanical metamaterials through hybrid kirigami structures. Sci. Rep..

[bib26] Iniguez-Rabago A., Li Y., Overvelde J.T.B. (2019). Exploring multistability in prismatic metamaterials through local actuation. Nat. Commun..

[bib27] Isanaka B., Mukhopadhyay T., Varma R., Kushvaha V. (2022). On exploiting machine learning for failure pattern driven strength enhancement of honeycomb lattices. Acta Mater..

[bib28] Jin L., Forte A.E., Deng B., Rafsanjani A., Bertoldi K. (2020). Kirigami-inspired inflatables with programmable shapes. Adv. Mater..

[bib29] Krishnaswamy J.A., Buroni F.C., Melnik R., Rodriguez-Tembleque L., Saez A. (2020). Design of polymeric auxetic matrices for improved mechanical coupling in lead-free piezocomposites. Smart Mater. Struct..

[bib30] Lakes R. (1987). Foam structures with a negative Poisson’s ratio. Science.

[bib31] Lakes R., Elms K. (1993). Indentability of conventional and negative Poisson’s ratio foams. J. Compos. Mater..

[bib32] Li L., Ma C., Kong Q., Li W., Zhang Y., Ge S., Yan M., Yu J. (2014). A 3d origami electrochemical immunodevice based on a au@pd alloy nanoparticle-paper electrode for the detection of carcinoembryonic antigen. J. Mater. Chem. B.

[bib33] Liu K., Paulino G.H. (2017). Nonlinear mechanics of non-rigid origami: an efficient computational approach†. Proc. Math. Phys. Eng. Sci..

[bib34] Liu K., Paulino G.H. (2016). Proc. Of the IASS Annual Symp (Spatial Structures in the 21st Century, Tokyo, Japan, 26–30 September).

[bib35] Liu Y., Hu H. (2010). A review on auxetic structures and polymeric materials. Sci. Res. Essays.

[bib36] Ma J., Song J., Chen Y. (2018). An origami-inspired structure with graded stiffness. Int. J. Mech. Sci..

[bib37] Masters I., Evans K. (1996). Models for the elastic deformation of honeycombs. Compos. Struct..

[bib38] Meza L.R., Phlipot G.P., Portela C.M., Maggi A., Montemayor L.C., Comella A., Kochmann D.M., Greer J.R. (2017). Reexamining the mechanical property space of three-dimensional lattice architectures. Acta Mater..

[bib39] Mukhopadhyay T., Adhikari S. (2016). Effective in-plane elastic properties of auxetic honeycombs with spatial irregularity. Mech. Mater..

[bib40] Mukhopadhyay T., Adhikari S. (2016). Free-vibration analysis of sandwich panels with randomly irregular honeycomb core. J. Eng. Mech..

[bib41] Mukhopadhyay T., Adhikari S. (2017). Effective in-plane elastic moduli of quasi-random spatially irregular hexagonal lattices. Int. J. Eng. Sci..

[bib42] Mukhopadhyay T., Adhikari S. (2017). Stochastic mechanics of metamaterials. Compos. Struct..

[bib43] Mukhopadhyay T., Adhikari S., Alu A. (2019). Probing the frequency-dependent elastic moduli of lattice materials. Acta Mater..

[bib44] Mukhopadhyay T., Adhikari S., Alu A. (2019). Theoretical limits for negative elastic moduli in subacoustic lattice materials. Phys. Rev. B.

[bib45] Mukhopadhyay T., Ma J., Feng H., Hou D., Gattas J.M., Chen Y., You Z. (2020). Programmable stiffness and shape modulation in origami materials: emergence of a distant actuation feature. Appl. Mater. Today.

[bib46] Mukhopadhyay T., Mahata A., Adhikari S., Asle Zaeem M. (2018). Probing the shear modulus of two-dimensional multiplanar nanostructures and heterostructures. Nanoscale.

[bib47] Mukhopadhyay T., Naskar S., Adhikari S. (2020). Anisotropy tailoring in geometrically isotropic multi-material lattices. Extreme Mechanics Letters.

[bib48] Overvelde J.T.B., Weaver J.C., Hoberman C., Bertoldi K. (2017). Rational design of reconfigurable prismatic architected materials. Nature.

[bib49] Paik J., Kramer R., Wood R. (2011). IEEE/RSJ International Conference on Intelligent Robots and Systems.

[bib50] Prall D., Lakes R. (1997). Properties of a chiral honeycomb with a Poisson’s ratio of—1. Int. J. Mech. Sci..

[bib51] Saxena K.K., Das R., Calius E.P. (2016). Three decades of auxetics research- materials with negative Poisson’s ratio: a review. Adv. Eng. Mater..

[bib52] Scarpa F., Ciffo L.G., Yates J.R. (2003). Dynamic properties of high structural integrity auxetic open cell foam. Smart Mater. Struct..

[bib53] Schenk M., Guest S., Yim M. (2011). Origami5, A K Peters.

[bib54] Seepersad C.C., Kumar R.S., Allen J.K., Mistree F., Mcdowell D.L. (2004). Multifunctional design of prismatic cellular materials. J. Computer-Aided Mater. Des..

[bib55] Shi X., Zhu Y., Fan X., Wu H.A., Wu P., Ji X., Chen Y., Liang J. (2022). An auxetic cellular structure as a universal design for enhanced piezoresistive sensitivity. Matter.

[bib56] Silverberg J.L., Evans A.A., McLeod L., Hayward R.C., Hull T., Santangelo C.D., Cohen I. (2014). Using origami design principles to fold reprogrammable mechanical metamaterials. Science.

[bib57] Singh A., Mukhopadhyay T., Adhikari S., Bhattacharya B. (2022). Active multi-physical modulation of Poisson’s ratios in composite piezoelectric lattices: on-demand sign reversal. Compos. Struct..

[bib58] Singh A., Mukhopadhyay T., Adhikari S., Bhattacharya B. (2022).

[bib59] Smith C.W., Wootton R.J., Evans K.E. (1999). Interpretation of experimental data for Poisson’s ratio of highly nonlinear materials. Exp. Mech..

[bib60] Tang Y., Yin J. (2017). Design of cut unit geometry in hierarchical kirigami-based auxetic metamaterials for high stretchability and compressibility. Extreme Mechanics Letters.

[bib61] Waitukaitis S., Menaut R., Chen B.G.g., Van Hecke M. (2015). Origami multistability: from single vertices to metasheets. Phys. Rev. Lett..

[bib62] Wang H., Zhao D., Jin Y., Wang M., Mukhopadhyay T., You Z. (2020). Modulation of multi-directional auxeticity in hybrid origami metamaterials. Appl. Mater. Today.

[bib63] Wang L., Zhu S., Wang B., Tan X., Zou Y., Chen S., Li S. (2021). Latitude-and-longitude-inspired three-dimensional auxetic metamaterials. Extreme Mechanics Letters.

[bib64] Wang Y., Zhao W., Wang H., Liu Z. (2018). A bio-inspired novel active elastic component based on negative Poisson’s ratio structure and dielectric elastomer. Smart Mater. Struct..

[bib65] Wojciechowski K. (1987). Constant thermodynamic tension Monte Carlo studies of elastic properties of a two-dimensional system of hard cyclic hexamers. Mol. Phys..

[bib66] Wojciechowski K. (1989). Two-dimensional isotropic system with a negative Poisson ratio. Phys. Lett..

[bib67] Wu X., Jin Y., Khelif A., Zhuang X., Rabczuk T., Djafari-Rouhani B. (2021). Topological surface wave metamaterials for robust vibration attenuation and energy harvesting. Mech. Adv. Mater. Struct..

[bib68] Wu X., Wen Z., Jin Y., Rabczuk T., Zhuang X., Djafari-Rouhani B. (2021). Broadband Rayleigh wave attenuation by gradient metamaterials. Int. J. Mech. Sci..

[bib69] Yang L., Harrysson O., West H., Cormier D. (2015). Mechanical properties of 3d re-entrant honeycomb auxetic structures realized via additive manufacturing. Int. J. Solid Struct..

[bib70] Yasuda H., Yang J. (2015). Reentrant origami-based metamaterials with negative Poisson’s ratio and bistability. Phys. Rev. Lett..

[bib71] Yu X., Zhou J., Liang H., Jiang Z., Wu L. (2018). Mechanical metamaterials associated with stiffness, rigidity and compressibility: a brief review. Prog. Mater. Sci..

[bib72] Zhai Z., Wu L., Jiang H. (2021). Mechanical metamaterials based on origami and kirigami. Appl. Phys. Rev..

[bib73] Zheng X., Lee H., Weisgraber T.H., Shusteff M., DeOtte J., Duoss E.B., Kuntz J.D., Biener M.M., Ge Q., Jackson J.A. (2014). Ultralight, ultrastiff mechanical metamaterials. Science.

[bib74] Zschernack C., Wadee M.A., Völlmecke C. (2016). Nonlinear buckling of fibre-reinforced unit cells of lattice materials. Compos. Struct..

