# Supplementary material for: Kirigami-inspired metamaterials for programming constitutive laws: Mixed-mode multidirectional auxeticity and contact-induced stiffness modulation
Source: iScience. 2022 Dec 8;25(12):105656. doi: 10.1016/j.isci.2022.105656 (PMC9801249; doi:10.1016/j.isci.2022.105656)
Supplement: Document S1. Figures S1–S11 [file mmc1.pdf]

iScience, Volume *25*

Supplemental information

Kirigami-inspired metamaterials for programming constitutive laws: Mixed-mode multidirectional auxeticity and contact-induced stiffness modulation

Aryan Sinha and Tanmoy Mukhopadhyay

Supplementary Material

Kirigami-inspired metamaterials for programming constitutive laws: Mixed-mode multi-directional auxeticity and contact-induced stiffness modulation

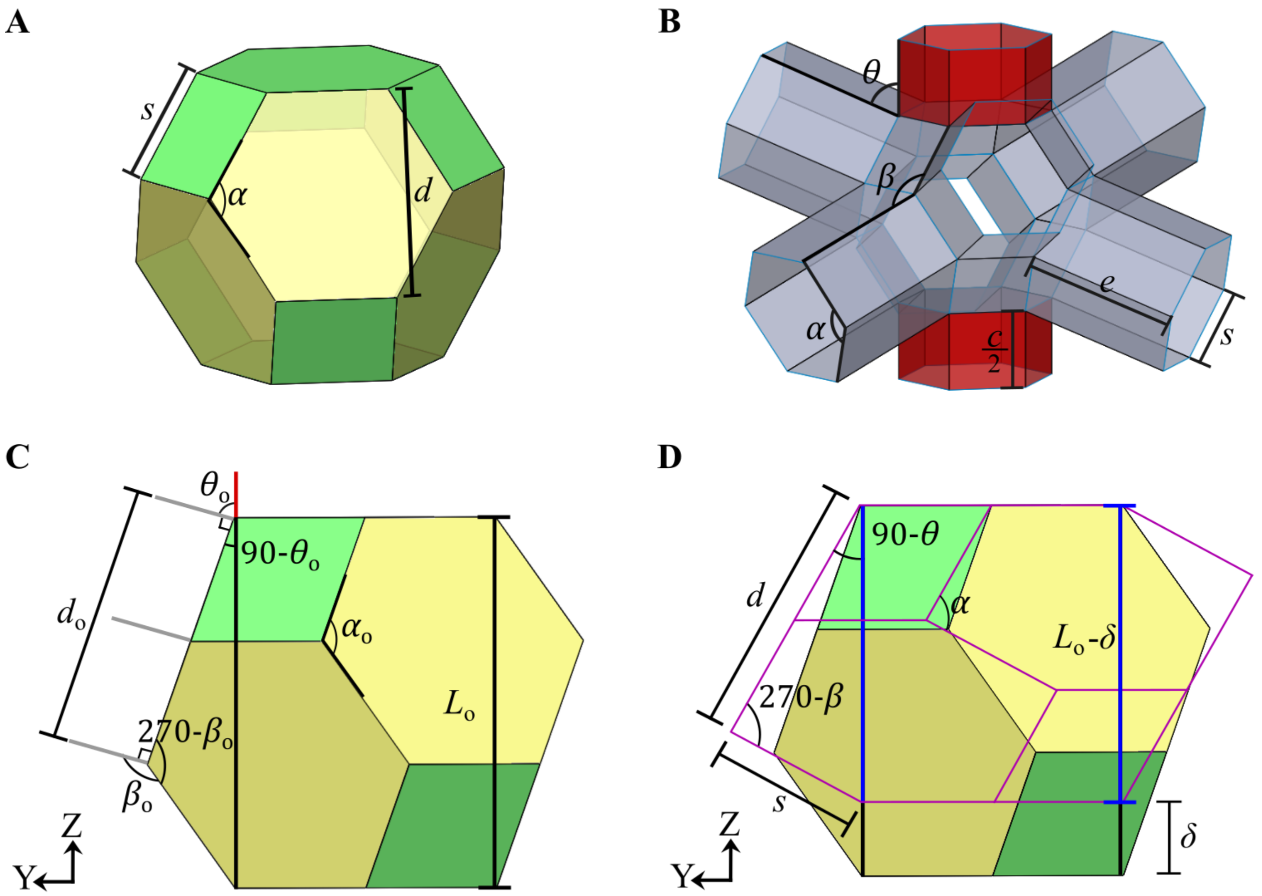

Figure S1: Truncated octahedron model of the proposed unit-cell. (A) Truncated octahedron of side length s and distance between longitudinally opposite sides of a hexagonal base defined as d. The yellow faces are replaced by extrusions while the green faces are rigid. **(B)** Geometry of the proposed 'symmetric' unit-cell derived from a truncated octahedron. It can be geometrically defined by a side length (s), extrusion length (e) and connector length (c). **(C)** Side view (ZY Plane) of the undeformed state. **(D)** Side view (ZY Plane) of the wire-frame model, given a longitudinal deformation δ, superimposed on the undeformed model state. (Related to section 4, Method details, **STAR Methods**.)

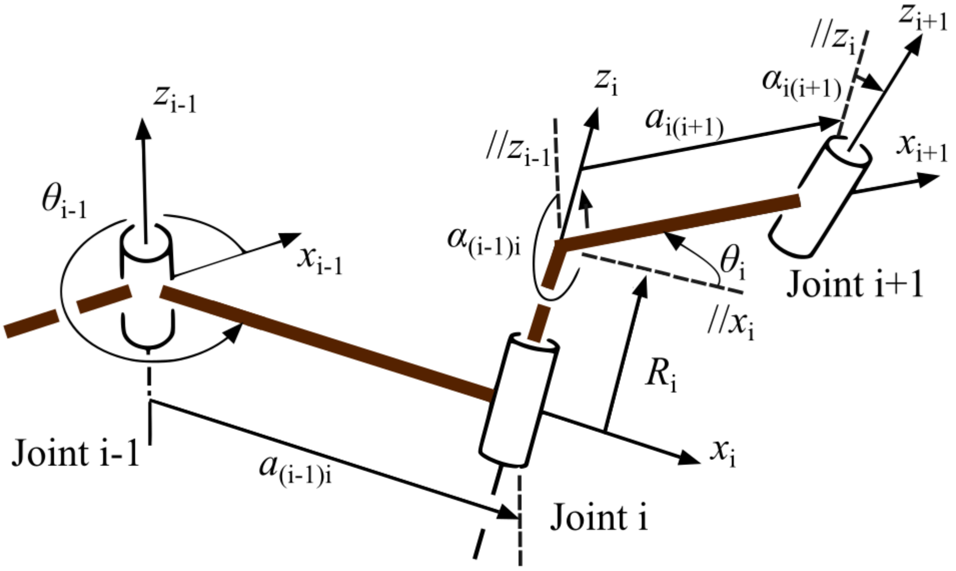

Figure S2: The D-H notation of adjacent links connected by revolute joints. (Related to section 4, Method details, **STAR Methods**.)

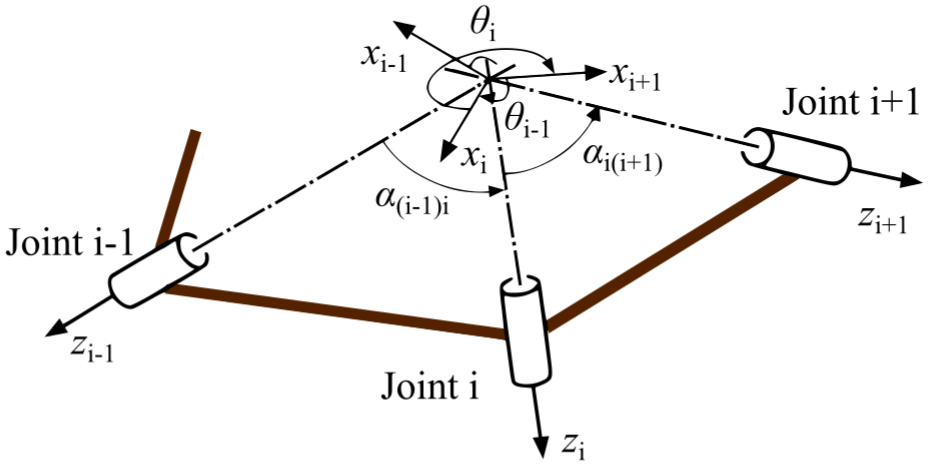

Figure S3: The D-H notation of a portion of a spherical linkage. (Related to section 4, Method details, **STAR Methods**.)

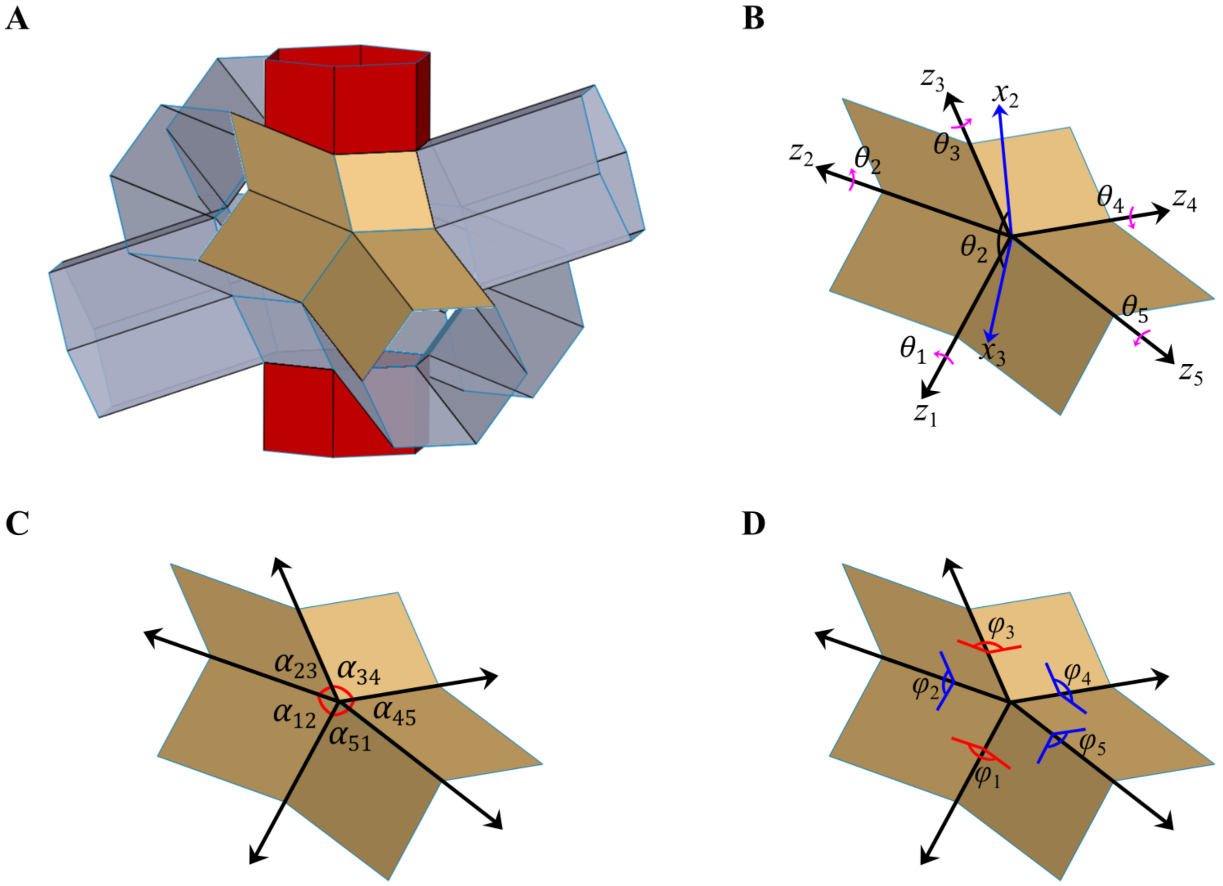

Figure S4: Kinematic analysis. (A) Proposed unit-cells with facets associated with the unit linkage loop highlighted. **(B)** Axes x_2, x_3, and kinematic variable θ_2 depicted along with the rotational z axes in the unit spherical linkage. **(C)** Angles of rotation α denoted for the rotational z axes in the unit spherical linkage. **(D)** Dihedral Angles marked on valley creases (in red) and mountain creases (in black) denoted on the unit linkage loop. (Related to section 4, Method details, **STAR Methods**.)

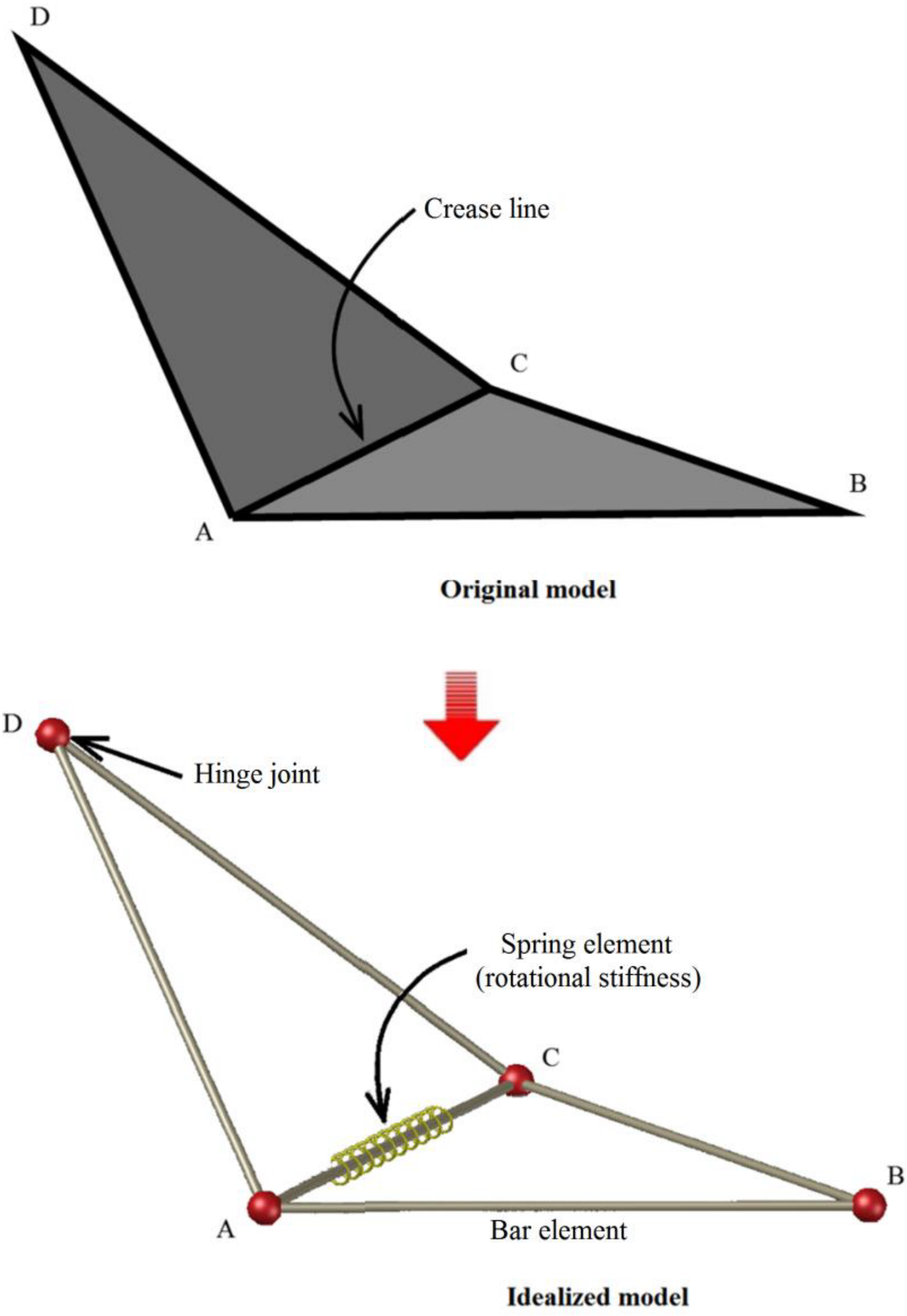

Figure S5: Bar and hinge model for idealizing the structural behaviour of origami. (Related to section 5, Method details, **STAR Methods**.)

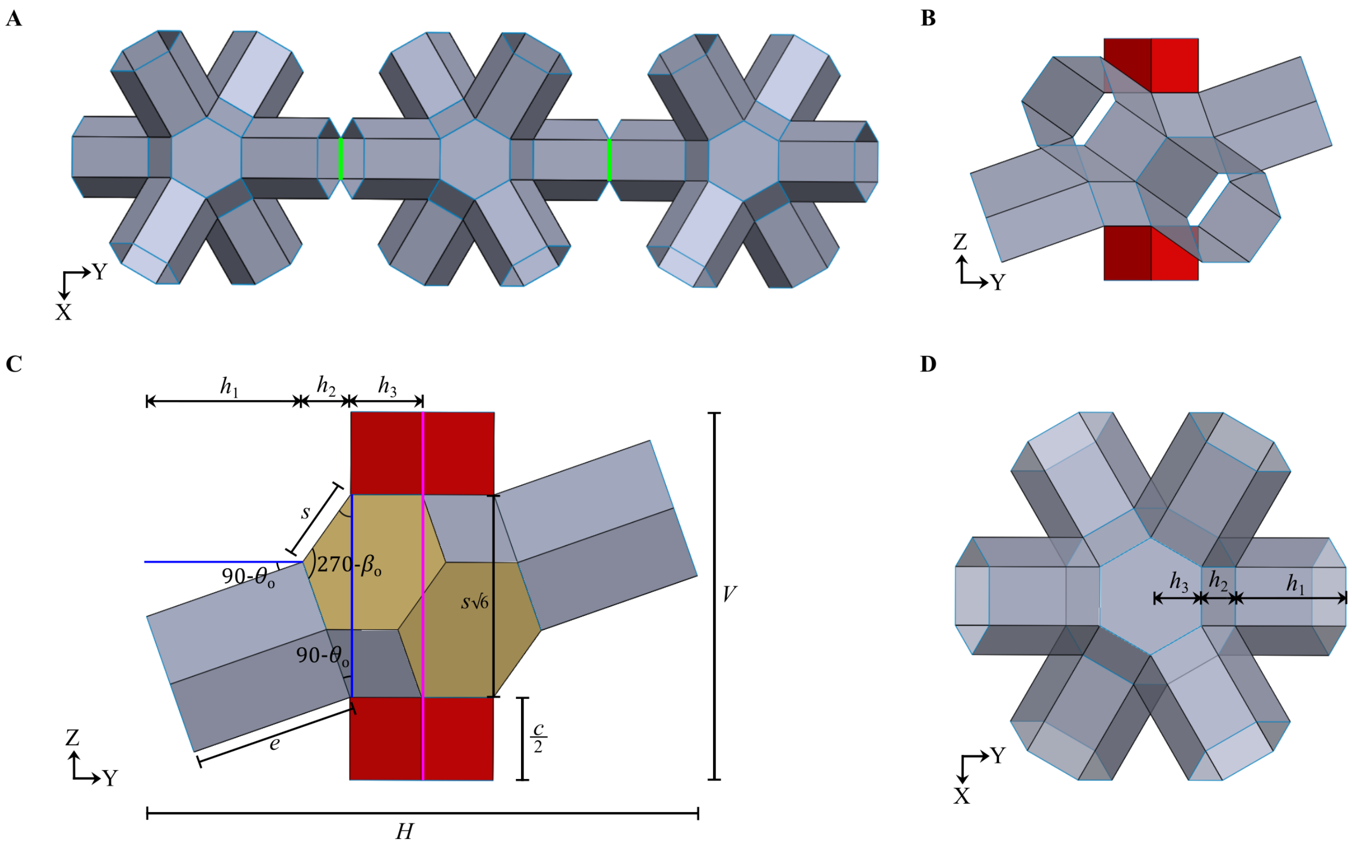

Figure S6: In-Plane Poisson's Ratio. **(A)** Top view (XY Plane) of the metamaterial with in-plane hinged connections marked in green. **(B)** Side view (YZ Plane) of a unit-cell. **(C)** Side view of a unit-cell with the two out-of-plane extrusions replaced with hexagonal faces (in yellow) at their bases for better visualization. **(D)** Top view (XY Plane) of a unit-cell. (Related to section 6, Method details, **STAR Methods**.)

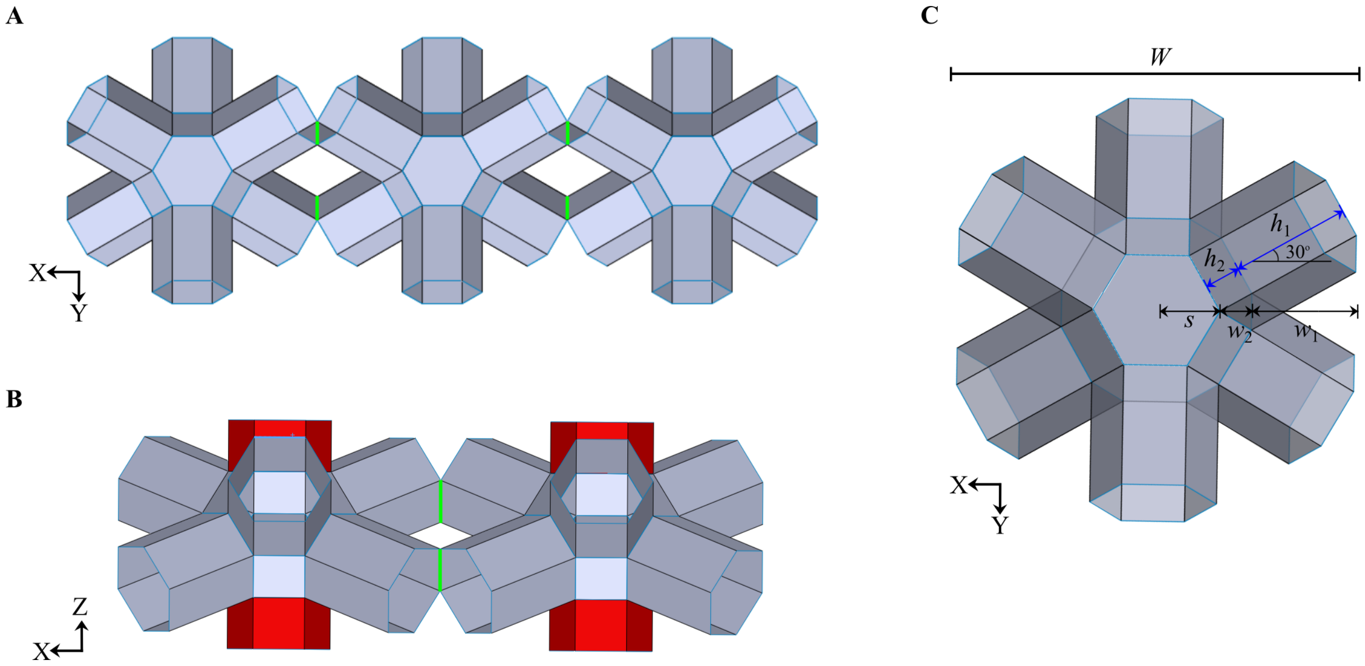

Figure S7: Out-of-Plane Poisson's Ratio. (A) Top view (XY Plane) of the metamaterial with out-of-plane hinged connections marked in green. **(B)** Side view (YZ Plane) of a pair of unit-cells attached through hinged connections (in green). **(C)** Top view (XY Plane) of a unit-cell. (Related to section 6, Method details, **STAR Methods**.)

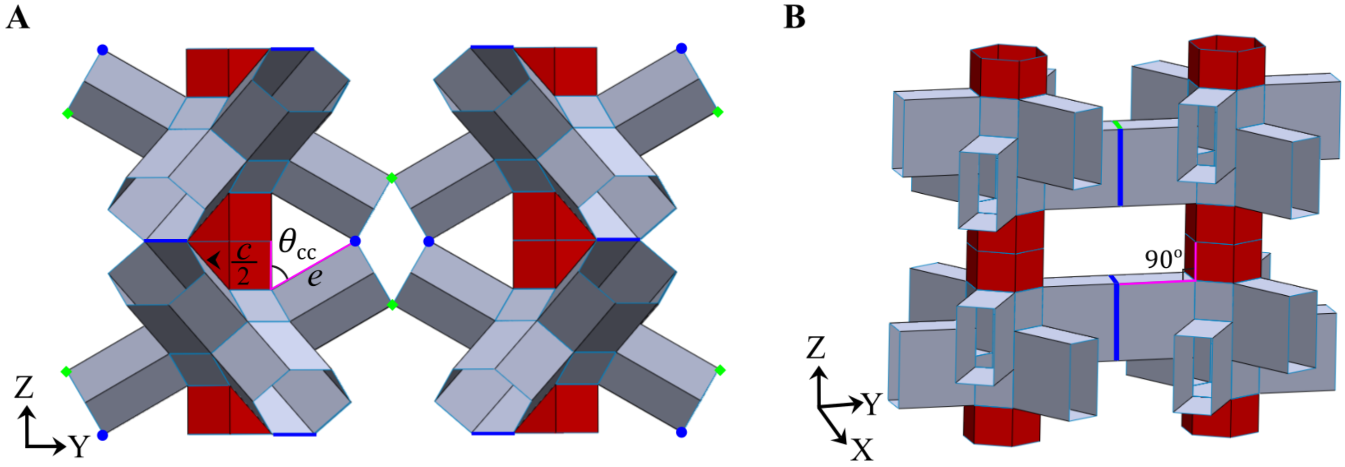

Figure S8: Microstructural geometry upon contact. (A) Geometric configuration of unit-cells at a state of contact in longitudinal compressive loading. The edges in contact are marked in blue while the hinged connections are marked in green. **(B)** Geometric configuration of unit-cells at a state of contact in longitudinal tensile loading. The edges in contact are marked in blue while the hinged connections are marked in green. (Related to section 7, Method details, **STAR Methods**.)

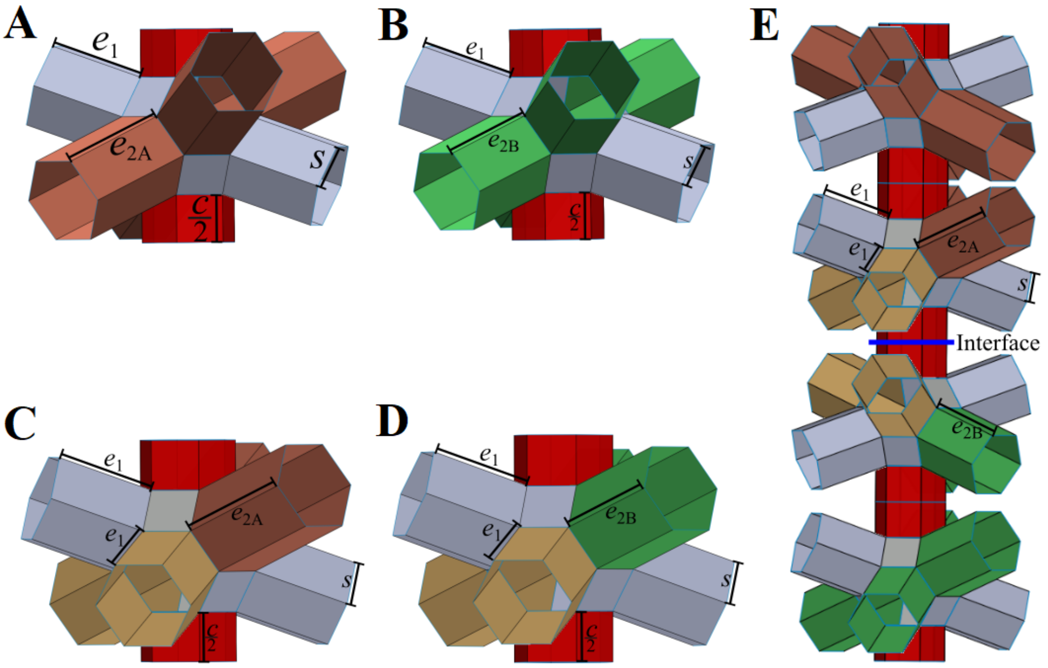

Figure S9: Asymmetric unit-cells. **(A)** 'Asymmetric-I' unit-cell with side length (s) = 1, connector length (c) = 2, In-plane extrusion length (e_1) = 2 and, Out-of-plane extrusion length (e_{2A}) = 2.6. The in-plane extrusions are depicted in grey while the out-of-plane extrusions are depicted in brown. **(B)** 'Asymmetric-I' unit-cell with side length (s) = 1, connector length (c) = 2, In-plane extrusion length (e_1) = 2 and, Out-of-plane extrusion length (e_{2B}) = 2.5. The in-plane extrusions are depicted in grey while the out-of-plane extrusions are depicted in green. **(C)** 'Asymmetric-II' unit-cell with side length (s) = 1, connector length (c) = 2, In-plane extrusion length (e_1) = 2, downward-pointing out-of-plane extrusion length (e_1) = 2 and, upward-pointing out-of-plane extrusion length (e_{2A}) = 2.6. The in-plane extrusions are depicted in grey, the downward-pointing out-of-plane extrusions are depicted in yellow and, the upward-pointing out-of-plane extrusions are depicted in brown. **(D)** 'Asymmetric-II' unit-cell with side length (s) = 1, connector length (c) = 2, In-plane extrusion length (e_1) = 2, downward-pointing out-of-plane extrusion length (e_1) = 2 and, upward-pointing out-of-plane extrusion length (e_{2B}) = 2.5. The in-plane extrusions are depicted in grey, the downward-pointing out-of-plane extrusions are depicted in yellow and, the upward-pointing out-of-plane extrusions are depicted in green. **(E)** Depiction of the interface that would form between two 'sets' as defined in Figure 4 of the main paper. The top-most unit-cell represents the 'set' formed with out-of-plane extrusion length (e_{2A}) = 2.6. The bottom-most unit-cell represents the 'set' formed with out-of-plane extrusion length (e_B) = 2.5. 'Asymmetric-II' unit-cells from figures C-D are added in-between to ensure proper edge-to-edge contact at the interface (depicted in blue) of these two 'sets.' (Related to section 7, Method details, **STAR Methods**.)

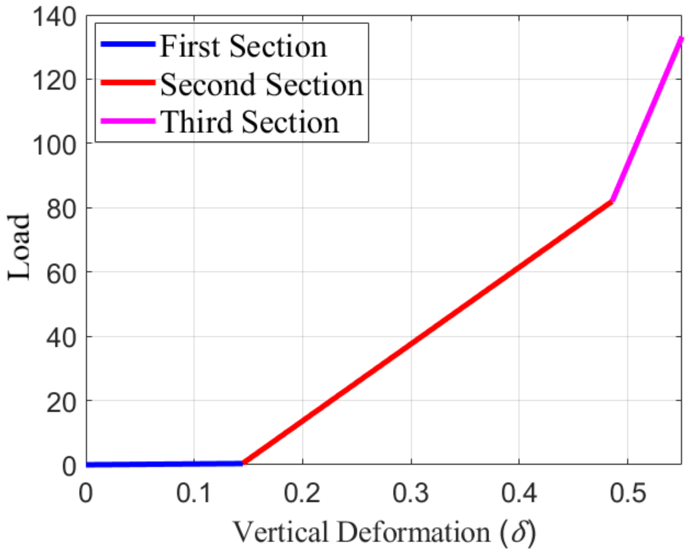

Figure S10: Constitutive relation for 'asymmetric-I' unit-cell under compressive loading. (Related to section 8, Method details, **STAR Methods**.)

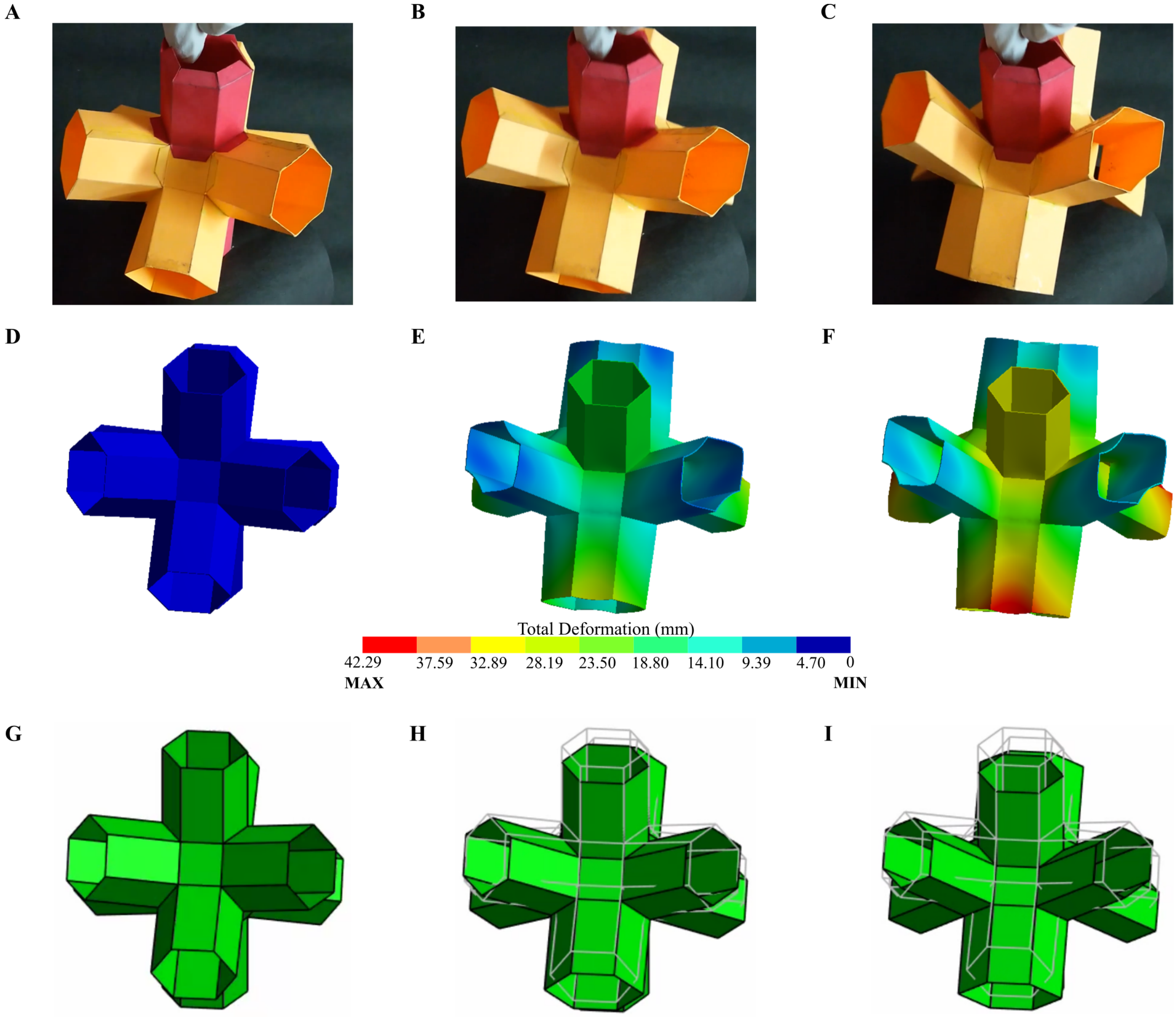

Figure S11: Qualitative comparison of the deformation behavior considering a symmetric unit-cell through physical prototypes, finite element simulation and idealized structural simulation. Here we have considered three stages of deformation for each of the analysis (geometric dimensions: $s = 20$ mm, $e = 40$ mm, $c = 40$ mm). Figures A, D and G represent the unreformed stage. Figures B, E and H represent the deformed states at stage 2. Figures C, F and I represent the deformed states at stage 3. Note that deformation at stage 3 is more than stage 2. The exact magnitudes of deformation can be understood from the presented color bar. **(A)** Physical prototype of undeformed symmetric unit-cell. **(B)** Physical prototype under intermediate longitudinal deformation at stage 2. **(C)** Physical prototype at larger deformed state (stage 3). **(D)** FE model of undeformed symmetric unit-cell. **(E)** FE model under intermediate longitudinal deformation (stage 2).**(F)** FE model at larger deformed state (stage 3).**(G)** Idealized structural simulation model of undeformed symmetric unit-cell. **(H)** Idealized structural simulation model under intermediate longitudinal deformation (stage 2). **(I)** Idealized structural simulation model at larger deformed state (stage 3). Grey wireframe represents the undeformed geometry for reference. (Related to section 9, Method details, **STAR Methods**.)
